# Complete mitochondrial genome of the lappet moth, *Kunugia undans* (Lepidoptera: Lasiocampidae): genomic comparisons among macroheteroceran superfamilies

**DOI:** 10.1590/1678-4685-GMB-2016-0298

**Published:** 2017-07-31

**Authors:** Min Jee Kim, Jun Seong Jeong, Jong Seok Kim, Su Yeon Jeong, Iksoo Kim

**Affiliations:** Department of Applied Biology, College of Agriculture & Life Sciences, Chonnam National University, Gwangju, Republic of Korea

**Keywords:** Kunugia undans, mitochondrial genome, Lasiocampoidea, Macroheterocera

## Abstract

The mitochondrial genome (mitogenome) characteristics of the monotypic Lasiocampoidea are largely unknown, because only limited number of mitogenomes is available from this superfamily. In this study, we sequenced the complete mitogenome of the lappet moth, *Kunugia undans* (Lepidoptera: Lasiocampidae) and compared it to those of Lasiocampoidea and macroheteroceran superfamilies (59 species in six superfamilies). The 15,570-bp *K. undans* genome had one additional *trnR* that was located between *trnA* and *trnN* loci and this feature was unique in Macroheterocera, including Lasiocampoidea. Considering that the two *trnR* copies are located in tandem with proper secondary structures and identical anticodons, a gene duplication event might be responsible for the presence of the two tRNAs. Nearly all macroheteroceran species, excluding Lasiocampoidea, have a spacer sequence (1–34 bp) at the *trnS*_*2*_ and *ND1* junction, but most lasiocampid species, including *K. undans,* have an overlap at the *trnS*_*2*_ and *ND1* junction, which represents a different genomic feature in Lasiocampoidea. Nevertheless, a TTAGTAT motif, which is typically detected in Macroheterocera at the *trnS*_*2*_ and *ND1* junction, was also detected in all Lasiocampoidea. In summary, the general mitogenome characteristics of Lasiocampoidea did not differ greatly from the remaining macroheteroceran superfamilies, but it did exhibit some unique features.

## Introduction

The typical metazoan mitochondrial genome (mitogenome) consists of 13 protein-coding genes (PCGs), 22 tRNAs, two rRNAs, and a major non-coding sequence referred to as the A+T-rich region. The characteristic features of the mitogenome (*e.g.*, fast evolution, low recombination rates, and multiple copies per cell) are considered beneficial in several biological fields (Cameron, 2014). In particular, whole mitogenome sequences have been utilized for phylogenic analyses of several insect lineages (Dowton *et al.*, 1997; Kim *et al.*, 2011; Lu *et al.*, 2013; Mao *et al.*, 2014, 2015; Timmermans *et al.*, 2014), and genomic characteristics have also been scrutinized to understand phylogenetic and evolutionary features of given taxonomic groups (Cameron and Whiting*,* 2008; Wan *et al.*, 2013; Kim *et al.*, 2014).

Mitogenome sequences in insects have been compiled in nearly 1,000 species that represent all insect orders and the Lepidoptera. As one of the four most species-rich insect orders, Lepidoptera is represented by 338 mitogenomes in GenBank (last visited on August 14, 2016), including 37 nearly complete sequences from 23 superfamilies. Among these, the monotypic Lasiocampoidea is represented by four species in two genera. Considering that the monotypic superfamily consists of 1,952 species with five subfamilies (van Nieukerken *et al.*, 2011), mitogenome sequences from additional diverse taxonomic groups could be required for mitogenome-based phylogenetic studies. In fact, recent large-scale mitogenome-based lepidopteran phylogenies only included a single genus or a single species (Timmermans *et al.*, 2014; Ramírez-Ríos *et al.*, 2016).

The lappet moth, *Kunugia undans* (Walker) (Lepidoptera: Lasiocampidae), is distributed in South Korea (excluding the far eastern Ulleungdo Island), far eastern Russia, Japan, and Australia (Park *et al.*, 1999; Shin, 2001). In Korea, adults are found from September to October, eggs then overwinter, and larvae hatch in the spring (Park *et al.*, 1999). Its host plants are *Castanea crenata* S. et Z., *Quercus acutissima* Carr., *Quercus variabilis* Bl. in Fagaceae, and *Malus pumila* var. *dulcissima* Koidz. in Rosaceae (Park *et al.*, 1999). Variations in size, coloration, and lines on the wings are present. The wingspan of the species is 56–65 mm in males and 79–92 mm in females, and forewings have a small white spot at the medial cell (Shin, 2001).

In this study, we determined the complete mitogenome sequence of the lappet moth *K. undans*, adding a new mitogenome sequence of a previously unreported genus of Lasiocampoidea. The genomic characteristics of the sequence were compared to those of other lasiocampid species in terms of genome structure, genomic arrangement, nucleotide composition, codon usage, etc. Furthermore, to better understand the evolutionary characteristics of the Lasiocampoidea, including *K. undans,* the mitogenome sequences were compared to the representatives of the Macroheterocera clade, to which Lasiocampoidea belongs.

## Materials and Methods

### DNA extraction, PCR and sequencing

An adult *K. undans* was collected from Shinan-gun in Jeollanamdo Province in Korea (34°3'60″ N, 125°6'50″ E) in 2009. After collection in the field, the sample was prepared as a dried specimen and deposited at Chonnam National University, Gwangju, Korea under the accession code KTOL-Bom-27. DNA was extracted from the hind legs using a Wizard Genomic DNA Purification Kit, in accordance with the manufacturer's instructions (Promega, Madison, WI, USA). For whole mitogenome sequencing, primers that amplify three long overlapping fragments (LF1 from *COI* and *ND4*, LF2 from *ND5* to *lrRNA*, and LF3 from *lrRNA* to *COI*) were adapted from Kim *et al.* (2012).

Three long fragments (LFs) were amplified using LA Taq^TM^ (Takara Biomedical, Tokyo, Japan) under the following conditions: 96 °C for 2 min; 30 cycles of 98 °C for 10 sec and 48 °C for 15 min; and a final extension step of 72 °C for 10 min. Using the LFs as templates, 26 overlapping short fragments (SF) were amplified using the primers adapted from Kim *et al.* (2012) and AccuPower^®^ PCR Pre-Mix (Bioneer, Daejeon, Korea). The PCR conditions for SFs were as follows: denaturation for 5 min at 94 °C; 35 cycles of 1 min denaturation at 94 °C; 1 min annealing at 48–51 °C; 1 min extension at 72 °C; and a final extension of 7 min at 72 °C. Primers used to amplify and sequence the LFs and SFs are presented in Table S1. DNA sequencing was conducted using the ABI PRISM® BigDye^®^ Terminator v3.1 Cycle Sequencing Kit and an ABI PRISMTM 3100 Genetic Analyzer (PE Applied Biosystems, Foster City, CA, USA). All products were sequenced from both directions.

### Gene annotation

Individual SF sequences were assembled into the complete mitogenome using Seqman software (DNASTAR, Madison, Wisconsin, USA). Identification, boundary delimitation, and secondary structure folding of tRNAs were performed using tRNAscan-SE 1.21 with the search mode set as default, the Mito/Chloroplast as the searching source, the genetic code of invertebrate mitogenomes for tRNA isotype prediction, and a cove score cut-off of 1 (Lowe and Eddy, 1997). Twenty-one tRNAs were detected based on these parameters. However, *trnS*_*1*_, which has a truncated DHU arm, was detected using a hand-drawn secondary structure in conjunction with an alignment of the predicted *trnS*_*1*_ regions of other lasiocampid species, and the anticodon was given particular consideration (Timmermans *et al.*, 2014; Qin *et al.*, 2015; Kim *et al.*, 2016). Individual PCGs were identified, and a boundary was delimited using the blastx and tblastn programs in BLAST (http://blast.ncbi.nlm.nih.gov/BLAST.cgi). With the aid of sequences from other lasiocampid species, the start and stop codons of PCGs were confirmed using MAFFT ver. 6 (Katoh *et al.*, 2002). Two rRNAs and the A+T-rich region were identified and delimited using the nucleotide blast algorithm in Blast, and it was further confirmed with the alignment of mitochondrial rRNA genes and sequences of the A+T-rich region of other lasiocampid species using MAFFT ver. 6.

### Comparative analysis

For the comparative analysis of the *K. undans* mitogenome, available lasiocampid species and one species from each genus of the macroheteroceran superfamily were downloaded from either GenBank or AMiGA (Feijao *et al.*, 2006), resulting in 11 mitogenome sequences from four Lasiocampidae species (including *K. undans*) and 48 species from five macroheteroceran superfamilies (Bombycoidea, Geometroidea, Noctuoidea, Drepanoidea, and Mimallonoidea). The nucleotide sequences of the PCGs were translated based on the invertebrate genetic code for mitochondrial DNA (mtDNA). Codon usage and nucleotide composition were determined by MEGA 6 (Tamura *et al.*, 2013), and gene overlap and intergenic-space sequences were hand-counted. The A/T content of each gene, whole genome, and each codon position of the PCGs were calculated with DNASTAR (Madison, USA) (Burland, 2000). The *K. undans* sequence data were deposited to GenBank under accession no. KX822016.

## Results and Discussion

### Mitogenome organization and composition

The mitogenome size of *K. undans* is 15,570 bp, and is slightly larger than that of any other lasiocampid species, which range in size from 15,407 bp in *Dendrolimus punctatus* (KJ913814) to 15,552 bp in *Apatelopteryx phenax* (KJ508055) (Table 1). *K. undans* contains 3,735 codons, excluding termination codons, and this number is the third largest in the sequenced Lasiocampoidea (next to *D. spectabilis* and *A. phenax*; Table 1). The size and codon counts of the lasiocampid species are well within the range found in macroheteroceran species, and no peculiarities associated with total size and codon count were detected in Lasiocampoidea (Table 1, Table S2).

**Table 1:** Characteristics of Lasiocampoidea mitogenomes.

Taxon	Size (bp)	A/T content (%)	PCG		srRNA		lrRNA		tRNA		A+T-rich region		GenBank accession no.	References
			No. codons^a^	AT%	Size (bp)	AT%	Size (bp)	AT%	Size (bp)	AT%	Size (bp)	AT%		
Lasiocampoidea														
Lasiocampidae														
*Kunugia undans*	**15,570**	**78.64**	**3,735**	**76.64**	**782**	**86.06**	**1,514**	**83.29**	**1,479**	**81.54**	**317**	**88.64**	**KX822016**	**This study**
*Apatelopteryx phenax*	15,552	80.33	3,736^b^	78.42	747	84.87	1,346	84.77	1,493	81.65	458	94.54	KJ508055	Timmermans *et al*., 2014
*Dendrolimus spectabilis*	15,409	79.45	3,724	77.56	777	85.20	1,454	83.84	1,469	81.21	320	92.50	KU558688	Kim *et al.*, 2016
*Dendrolimus spectabilis*	15,412	79.36	3,726	77.41	781	85.15	1,454	83.91	1,468	81.20	320	93.44	KJ913815	Qin *et al.*, 2015
*Dendrolimus spectabilis*	15,410	79.38	3,726	77.44	779	85.11	1,454	83.91	1,468	81.20	320	93.44	KJ913816	Qin *et al.*, 2015
*Dendrolimus spectabilis*	15,411	79.50	3,740	77.65	628	84.08	1,357	83.49	1,466	81.04	465	92.04	KM244678	Tang *et al.*, 2014
*Dendrolimus punctatus*	15,419	79.40	3,727	77.46	779	84.60	1,462	84.82	1,469	80.87	320	92.50	KJ913811	Qin *et al.*, 2015
*Dendrolimus punctatus*	15,418	79.46	3,727	77.51	779	84.98	1,462	84.82	1,469	81.01	320	92.50	KJ913812	Qin *et al.*, 2015
*Dendrolimus punctatus*	15,411	79.46	3,727	77.55	779	84.98	1,461	84.60	1,469	80.87	320	92.81	KJ913813	Qin *et al.*, 2015
*Dendrolimus punctatus*	15,407	79.38	3,727	77.50	780	84.74	1,452	84.44	1,469	80.80	320	91.88	KJ913814	Qin *et al.*, 2015
*Dendrolimus tabulaeformis*	15,411	79.53	3,726	77.63	778	84.70	1,459	84.72	1,469	81.01	320	92.81	KJ913817	Qin *et al.*, 2015
*Dendrolimus tabulaeformis*	15,409	79.40	3,726	77.44	779	84.98	1,456	84.62	1,468	81.06	320	92.81	KJ913818	Qin *et al.*, 2015

a Termination codons were excluded in the total codon count.

b Sequences include a few undetermined nucleotides.

**Figure 1:**
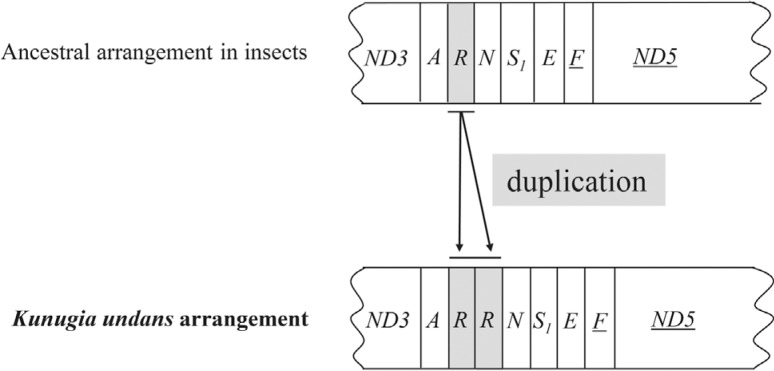
Schematic illustration of the gene arrangement with the duplicated *trnR* detected in *Kunugia undans*. Gene sizes are not drawn to scale. Gene names that are not underlined indicate a forward transcriptional direction, whereas underlined sequences indicate a reversed transcriptional direction. tRNAs are denoted by one-letter symbols in accordance with the IUPAC-IUB single-letter amino acid codes. The remaining genes and gene order configurations that are identical to ancestral insects are omitted.

Compared to the typical sets of genes and regions found in animal mitogenomes (13 PCGs, 22 tRNAs, 2 rRNA genes, and one non-coding A+T-rich region), the *K. undans* mitogenome contains one extra *trnR*, which is located in tandem to another *trnR* [referred to as *trnR* (A) for the copy located next to *trnA* and *trnR* (B) for the copy located next to *trnN*] between *trnA* and *trnN* (Figure 1). Pairwise sequence divergence between the two tRNAs was 10.94% (7 bp). Among lasiocampid species (data not shown), pairwise sequence divergence was 3.18-7.81% and 10.94% compared to *trnR* (A) and *trnR* (B), respectively, indicating that *trnR* (A) is more likely to be a functional copy, in that the sequence divergence range reflects the current taxonomic hierarchy. Nevertheless, both *trnR* copies have an identical anticodon (TCG) that is found in all other Lasiocampoidea (Table 2, Table S3), and they exhibit the proper secondary cloverleaf structure (Figure S1). Thus, the functionality of *trnR* (B) remains unknown. The tandem location of two *trnR* copies that exhibit proper secondary structures and an identical anticodon may indicate a gene duplication event rather than horizontal transfer (Higgs *et al.*, 2003). In Lepidoptera, *Coreana* *raphaelis* (Papilionoidea) was the first species reported to have 23 tRNA genes instead of the usual 22 because of a tandemly duplicated *trnS*_*1*_ between *trnN* and *trnE* (Kim *et al.*, 2006). *Ctenoptilum vasava* (Papilionoidea) was subsequently reported to have an extra *trnS*_*1*_ (Kim *et al.*, 2006; Hao *et al.*, 2012). However, the extra *trnR* found in the *K. undans* mitogenome is likely unique in Macroheterocera, in that our careful reexamination of all available lasiocampid species and all Macroheterocera did not reveal extra tRNAs (data not shown). Currently, the *K. undans* mitogenome is the only available *Kunugia* sequence, so whether this duplication event was species- or genus-specific is an intriguing question.

**Table 2:** Genomic summary of *Kunugia undans*.

Gene	Anticodon	Start codon	Stop codon	Nucleotide position (size)
*trnM*	CAT	-	-	1-68 (68)
*trnI*	GAT	-	-	72-135 (64)
*trnQ*	TTG	-	-	136-205 (70)
*ND2*		ATT	TAA	263-1276 (1014)
*trnW*	TCA	-	-	1275-1344 (70)
*trnC*	GCA	-	-	1337-1402 (66)
*trnY*	GTA	-	-	1412-1479 (68)
*COI*		CGA	T-tRNA	1500-3057 (1558)
*trnL*_*2*_	TAA	-	-	3058-3125 (67)
*COII*		ATA	T-tRNA	3125-3806 (682)
*trnK*	CTT	-	-	3807-3877 (71)
*trnD*	GTC	-	-	3879-3947 (69)
*ATP8*		ATC	TAA	3948-4109 (162)
*ATP6*		ATG	TAA	4103-4780 (678)
*COIII*		ATG	TAA	4787-5575 (790)
*trnG*	TCC	-	-	5578-5644 (67)
*ND3*		ATC	TAA	5645-5998 (354)
*trnA*	TGC	-	-	6003-6070 (68)
*trnR* (A)	TCG	-	-	6084-6147 (64)
*trnR* (B)	TCG	-	-	6175-6241 (67)
*trnN*	GTT	-	-	6242-6308 (67)
*trnS*_*1*_	GCT	-	-	6308-6375 (68)
*trnE*	TTC	-	-	6376-6440 (65)
*trnF*	GAA	-	-	6471-6537 (67)
*ND5*		ATT	T-tRNA	6538-8275 (1738)
*trnH*	GTG	-	-	8276-8343 (68)
*ND4*		ATG	TAG	8348-9682 (1335)
*ND4L*		ATG	TAG	9688-9981 (294)
*trnT*	TGT	-	-	9986-10050 (65)
*trnP*	TGG	-	-	10051-10115 (65)
*ND6*		ATA	TAA	10124-10654 (531)
*CytB*		ATG	TAA	10662-11807 (1146)
*trnS*_*2*_	TGA	-	-	11809-11875 (67)
*ND1*		ATG	TAA	11869-12825 (957)
*trnL*_*1*_	TAG	-	-	12827-12892 (66)
*lrRNA*		-	-	12893-14406 (1513)
*trnV*	TAC	-	-	14407-14471 (65)
*srRNA*		-	-	14472-15253 (782)
A+T–rich region		-	-	15254-15570 (317)

tRNA abbreviations follow the IUPAC-IUB three letter code.

-, not applicable.

The A/T nucleotide composition of the whole genome was 78.64% in *K. undans*, indicating biased A/T nucleotides, but it represents the lowest percentage detected in lasiocampid species (Table 1). Among macroheteroceran superfamilies, the A/T composition of the whole mitogenome in Lasiocampoidea is slightly lower than that of any other macroheteroceran superfamily (79.47% *vs* 80.23-80.79%), but the difference is slight (Table S2). The A/T content among *K. undans* genes varied between RNA (86.06% in *srRNA*, 83.29% in *lrRNA*, and 81.54% in tRNAs) and PCG (76.64%) genes, and the same trend was also found in other sequenced Macroheterocera, including Lasiocampoidea (Table 1, Table S2).

The *K. undans* gene arrangement is identical to that of other ditrysian Lepidoptera that exhibit the *trnM-trnI-* *trnQ* order (where the underline indicates a gene inversion) at the A+T-rich region and ND2 junction, with the exception of the duplicated *trnR* (Table 2; Kim *et al.*, 2011; Timmermans *et al.*, 2014; Park *et al.*, 2016; Zhao *et al.*, 2016). This arrangement is found in all sequenced Macroheterocera (Park *et al.*, 2016), including Lasiocampoidea (Table 2; Table S3). However, it differs from the ancestral *trnI-* *trnQ* *-trnM* order found in the majority of insects and the lepidopteran superfamilies Hepialoidea and Nepticuloidea, which are ancient, non-ditrysian lepidopteran groups (Cao *et al.*, 2012; Timmermans *et al.*, 2014). Thus, this tRNA rearrangement has been regarded as synapomorphy for Ditrysia. However, a new arrangement, *trnI-trnM-* *trnQ*, was reported from a butterfly species belonging to Nymphalidae in Papilionoidea (Xuan *et al.*, 2016). Therefore, the latter arrangement might represent an autapomorphy, in that no other congeneric species has the arrangement (Park *et al.*, 2016).

### Genes

Twelve of the 13 *K. undans* PCGs started with ATN, but *COI* started with an alternative CGA start codon, as observed in other moths (Figure S2). There is no typical start codon at the 5'-end of *trnY* and the intergenic spacer sequence located between *trnY* and *COI*, so CGA is the only possible start codon for *COI* in *K. undans*. The CGA start codon is found in all other sequenced macroheteroceran superfamilies, but some authors designate the typical ATN codon as the start codon for *COI* (Figure S2). This start codon has been reported to be highly conserved at the start region of *COI* in other Lepidoptera, and it was confirmed in a species of Lepidoptera based on expressed sequence tag data (Margam *et al.*, 2011; Kim *et al.*, 2014; Park *et al.*, 2016). Thus, the presence of a CGA start codon is now considered a synapomorphic trait in Lepidoptera, although some exceptions exist. The mitochondrial PCGs available for Lasiocampoidea, including *K. undans*, ended with TAA in the majority of PCGs, but they also infrequently ended with a single T (Table 2; Table S3). The TAG stop codon was uniquely used in *K. undans* for *ND4* and *ND4L*, while other lasiocampid species used a single T for *ND4* and TAA for *ND4L* (Table 2; Table S3). The incomplete termination codon is known to result in a complete TAA stop codon via posttranslational modifications that occur during the mRNA maturation process (Ojala *et al.*, 1981).

The biased A/T content was reflected in the form of codon usage. For instance, among the 64 available codons, the most frequently used codons [TTA (leucine), ATT (isoleucine), TTT (phenylalanine), and ATA (methionine)] accounted for 37.2% in *K. undans*, and this value was the lowest frequency detected in Lasiocampoidea (Table 3). These four codons are all comprised of A or T nucleotides, thus indicating the biased usage of A/T nucleotides in Lasiocampoidea PCGs, including *K. undans*. Other macroheteroceran superfamilies have also shown a similar pattern, revealing 39.1–40.7% in Bombycoidea, 37.5–40.4% in Geometroidea, 38.0–44.6% in Noctuoidea, 40.8–40.9% in Drepanoidea, and 39.3% in Mimallonoidea (Table S4).

**Table 3:** Frequency of the four most frequently used codons in Lasiocampoidea.

Species	Codon				Total
	TTA (L)	ATT (I)	TTT (F)	ATA (M)	
Lasiocampoidea					
Lasiocampidae					
*Kunugia undans*	**409/10.7**	**381/10.4**	**323/8.6**	**270/7.5**	**1383/37.2**
*Apatelopteryx phenax*	453/12.1	414/11.1	337/9.0	285/7.6	1489/39.8
*Dendrolimus spectabilis* (KU558688)	450/12.0	402/10.7	338/9.0	280/7.5	1470/39.2
*Dendrolimus spectabilis* (KJ913815)	449/12.1	399/10.7	331/8.9	281/7.5	1460/39.2
*Dendrolimus spectabilis* (KJ913816)	449/12.1	399/10.7	331/8.9	281/7.5	1460/39.2
*Dendrolimus spectabilis* (KM244678)	450/12.0	402/10.7	338/9.0	280/7.5	1470/39.2
*Dendrolimus punctatus* (KJ913811)	464/12.4	398/10.7	330/8.9	268/7.2	1460/39.2
*Dendrolimus punctatus* (KJ913812)	463/12.4	399/10.7	330/8.9	268/7.2	1460/39.2
*Dendrolimus punctatus* (KJ913813)	463/12.4	401/10.8	331/8.9	267/7.2	1462/39.3
*Dendrolimus punctatus* (KJ913814)	457/12.3	399/10.7	331/8.9	275/7.4	1462/39.3
*Dendrolimus tabulaeformis* (KJ913817)	461/12.4	401/10.8	331/8.9	269/7.2	1462/39.3
*Dendrolimus tabulaeformis* (KJ913818)	455/12.2	399/10.7	335/9.0	268/7.2	1457/39.1
Average	452/12.1	400/10.7	332/8.9	274/7.3	1458/39.1

The corresponding amino acids are located in parentheses. Values after the backslash indicate the percentage of corresponding codons.

The nucleotide composition of the 13 concatenated PCGs in the *K. undans* mitogenome was 33.5, 43.2, 11.8, and 11.5% for adenine, thymine, cytosine, and guanine, respectively, indicating A/T bias (Table 4). The base composition at each codon position of the *K. undans* PCGs indicated that the third codon position (86.5%) had a substantially higher A/T content than the first (72.6%) and second (70.4%) codon positions. A similar pattern was detected in other sequenced Lasiocampoidea, with averages of 77.6, 73.0, and 89.0 in the first, second, and third positions, respectively (Table 4).

**Table 4:** Codon position-based nucleotide composition of 13 concatenated Lasiocampoidea PCGs.

Species	Overall				1st codon position				2nd codon position				3rd codon position			
	A	T	C	G	A	T	C	G	A	T	C	G	A	T	C	G
Lasiocampoidea																
Lasiocampidae																
*Kunugia undans*	**33.5**	**43.2**	**11.8**	**11.5**	**37.6**	**35.0**	**11.0**	**16.0**	**22.4**	**48.0**	**16.0**	**13.4**	**40.5**	**46.0**	**8.5**	**5.2**
*Apatelopteryx phenax*	34.0	44.5	10.4	11.0	37.9	36.0	9.7	16.1	21.9	49.0	16.3	13.1	42.3	49.0	5.3	3.8
*Dendrolimus spectabilis* (KU558688)	33.5	44.1	11.0	11.4	36.9	36.0	10.1	16.6	21.7	48.0	16.6	13.3	41.2	46.0	8.3	4.8
*Dendrolimus spectabilis* (KJ913815)	33.4	44.0	11.1	11.5	36.9	36.0	10.0	16.7	21.7	48.0	16.6	13.4	41.7	47.0	6.6	4.5
*Dendrolimus spectabilis* (KJ913816)	33.4	44.0	11.1	11.5	36.9	36.0	10.0	16.7	21.7	48.0	16.6	13.4	41.7	47.0	6.5	4.5
*Dendrolimus spectabilis* (KM244678)	33.5	44.1	11.0	11.4	36.9	36.0	10.1	16.6	21.7	48.0	16.6	13.3	42.0	48.0	6.4	4.1
*Dendrolimus punctatus* (KJ913811)	33.5	43.9	11.1	11.5	36.9	36.0	10.0	16.7	21.7	48.0	16.6	13.5	41.9	47.0	6.6	4.2
*Dendrolimus punctatus* (KJ913812)	33.5	44.0	11.0	11.5	36.9	36.0	10.0	16.8	21.7	48.0	16.6	13.4	42.0	47.0	6.5	4.2
*Dendrolimus punctatus* (KJ913813)	33.6	44.0	11.0	11.4	37.0	36.0	10.0	16.7	21.7	48.0	16.6	13.4	42.0	47.0	6.4	4.2
*Dendrolimus punctatus* (KJ913814)	33.6	43.9	11.2	11.3	36.9	36.0	10.2	16.7	21.7	48.0	16.6	13.4	42.3	47.0	6.6	3.9
*Dendrolimus tabulaeformis* (KJ913817)	33.6	44.0	11.0	11.4	36.9	36.0	10.0	16.7	21.7	48.0	16.6	13.4	42.1	48.0	6.2	4.1
*Dendrolimus tabulaeformis* (KJ913818)	33.5	43.9	11.1	11.4	36.9	36.0	10.1	16.7	21.7	48.0	16.6	13.4	41.9	47.0	6.6	4.2
Average	33.6	44.0	11.1	11.4	37.1	35.9	10.1	16.6	21.8	48.1	16.5	13.4	41.8	47.2	6.7	4.3

Stop codons were excluded in the count.

Two rRNA genes in *K. undans*, *lrRNA* and *srRNA*, were of 1,514 and 782 bp, respectively, (Table 2), and the sizes of the two genes in *K. undans* were larger than those of any found in other lasiocampid species, which ranged from 1,346 bp (*A. phenax*) to 1,452 bp (*D. punctatus*) in *lrRNA* and 747 bp (*A. phenax*) to 780 bp (*D. punctatus*) in *srRNA* (Table S2). tRNA sizes ranged from 64 bp (*trnI*) to 71 bp (*trnK*) in *K. undans*, and similar size ranges were found in other sequenced lasiocampid species (Table 2; Table S3). All *K. undans* tRNAs possessed invariable lengths of 7 bp for the aminoacyl stem, 7 bp for the anticodon loop, and 5 bp for the anticodon stem (Figure S1), and most tRNA size variation resulted from length variations in the DHU and TΨC arms. For instance, *trnS*_*1*_ has an atypical cloverleaf secondary structure that lacked the DHU stem, but the remaining *K. undans* tRNAs formed the typical secondary cloverleaf structure (Figure S1). The aberrant *trnS*_*1*_ has been reported in many metazoan species, including insects (Garey and Wolstenholme, 1989; Wolstenholme, 1992). The DHU stem and loop are involved in tertiary interactions required for the proper folding and functioning of tRNA (Rich and RajBhandary, 1976). Thus, an atypical secondary structure may hamper the functionality of tRNA, but a nuclear magnetic resonance analysis from nematodes demonstrated that the aberrant *trnS*_*1*_ also was functionally similar to typical tRNAs based on structural adjustments required to ensure ribosome fitting (Ohtsuki *et al.*, 2002).

### The A+T-rich region

The length of the A+T-rich region in *K. undans* was 317 bp, and A/T nucleotides made up 88.64% of the sequence (Table 2). This region contained the highest A/T content of any region of the *K. undans* mitogenome (Table 1). Moreover, this region was the shortest in length, and it contained the least A/T nucleotides among lasiocampid species (Table 2, Table S3).

**Figure 2:**
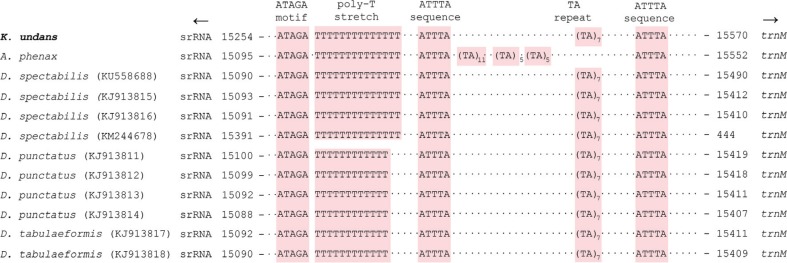
Schematic illustration of the A+T-rich region of Lasiocampoidea, including *Kunugia undans*. The colored nucleotides indicate conserved sequences such as the ATAGA motif, poly-T stretch, ATTTA sequence, and microsatellite-like TA repeat sequences. Dots between sequences indicate omitted sequences, and arrows indicate the transcriptional direction. Subscripts indicate the repeat number. GenBank accession numbers of the species represented by more than one mitogenome sequence are enclosed in parentheses.

The insect A+T-rich region harbors signals for replication and transcription initiation, so it is known to have conserved sequences in the region, which are in the form of conserved sequence blocks (Fauron and Wolstenholme, 1980; Clary and Wolstenholme, 1987; Saito *et al.*, 2005). In fact, previous studies revealed several conserved blocks in a substantial number of lepidopteran groups (Liao *et al.*, 2010; Kim *et al.*, 2014), and a search for the A+T-rich region of lasiocampid species (including *K. undans*) resulted in the detection of several conserved sequences (Figure 2). The first conserved sequence, which is located close to the 5'-end of the *srRNA*, is the ATAGA motif followed by a poly-T stretch of varying length. The *K. undans* A+T-rich region contained a 14-bp T stretch that was upstream of the 5'-end of the *srRNA* (Figure 2), and this poly-T stretch is well-conserved in all sequenced lasiocampid (ranging in size from 12 bp to 14 bp; Figure 2) and macroheteroceran species (Figure S3). Saito *et al.* (2005) previously reported for the *Bombyx mori* mitogenome the precise position of the replication origin for minor-strand mtDNA, which is immediately downstream of a poly-T stretch that is located upstream of the *srRNA* 5'-end. Thus, this poly-T stretch is thought to function as a possible recognition site for the initiation of replication of the minor mtDNA strand. Additionally, another conserved motif ATAGA is located immediately downstream of the poly-T stretch, and it is very well-conserved in all sequenced lasiocampid species, including *K. undans* (Figure 2) and macroheteroceran species (Figure S3). A previously suggested function of this motif is a regulatory role in conjunction with the poly-T stretch, but experimental data are required to support this hypothesis (Kim *et al.*, 2009). Excluding the previously described sequences, there are only a few additional conserved sequences in the A+T-rich region of lasiocampid [e.g., *K. undans* (Figure 2)] and macroheteroceran species (Figure S3), including two or more ATTTA sequences scattered in the A+T-rich region, a microsatellite-like TA repeat, and a poly-T stretch. Our careful reexamination of the A+T-rich regions of macroheteroceran species resulted in the detection of repeat sequences in several species, including two of each 55-bp and 24-bp repeats in *Bombyx huttoni* (Bombycoidea); six 26-bp and two 18-bp repeats in *Phthonandria atrilineata*, two 278-bp repeats in *Dysstroma truncata*, four 24-bp repeats in *Operophtera brumata* (Geometroidea), two 16-bp repeats in *Agrotis ipsilon*, and two 11-bp repeats in *Risoba prominens* (Noctuoidea) **(** Yang *et al.*, 2009; Timmermans *et al.*, 2014; Derks *et al.*, 2015; Wu *et al.*, 2015; Yang *et al.*, 2015; Peng *et al.*, 2016). Nevertheless, repeat sequences that were longer than 10 bp were not detected in sequenced lasiocampid species, including *K. undans*.

### Non-coding sequences

**Figure 3:**
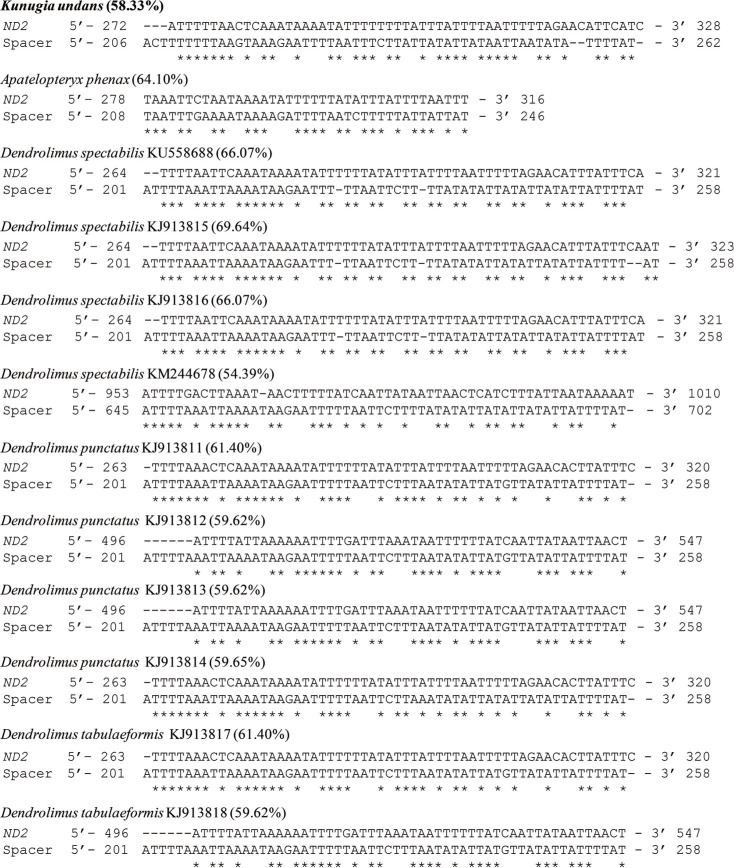
Alignment of the spacer sequence (located between *trnQ* and *ND2*) and the neighboring partial *ND2* of Lasiocampoidea, including *Kunugia undans*. Asterisks indicate consensus sequences in the alignment. Sequence homology between the spacer and *ND2* is shown in the parentheses next to the species name and GenBank accession numbers of species represented by more than one mitogenome sequences. The beginning and end nucleotide positions of the sequences are indicated.

Excluding the A+T-rich region, the *K. undans* mitogenome has non-coding sequences that total 172 bp (with a range of 1–57 bp) and spread over 17 regions (Table 2). Comparison of available lasiocampid species indicated that intergenic spacing patterns and sizes are largely consistent in Lasiocampoidea, including those of *K. undans*. In particular, the 57-bp spacer found at the *trnQ* and *ND2* junction (with a range of 39–58 bp) is consistently found in all lasiocampid species, including *K. undans* (Figure 3). The origin of this spacer region has previously been ascribed to the partial duplication and random loss of *ND2*, leaving the current length of the spacer sequence at the *trnQ* and *ND2* junction because the spacer exhibited sequence identity to the neighboring *ND2*, despite the fact that its non-coding nature may have allowed the spacer to diverge from the original *ND2* (Kim *et al.*, 2014). Regarding *K. undans*, the sequence identity of the spacer to the neighboring *ND2* was 58.33% (Figure 3) and over 50.60% in 59 species of macroheteroceran superfamilies (Figure S4).

Other relatively long spacer sequences were found in several regions of lasiocampid species, including *K. undans*, including those at the *trnY* and *COI* junction (20–34 bp), at the *trnA* and *trnR* junction (13–20 bp), at the *trnN* and *trnS*_*1*_ junction (11–21 bp, excluding *K. undan*s that has a 1-bp overlap), and at the *ND4* and *ND4L* junction (5–24 bp, excluding *A. phenax* that has a 5-bp overlap) (Table 2, Table S3). These spacer sequences are mainly composed of A/T nucleotides that are often found within multiple runs of either T or A nucleotides (data not shown). Sequence alignment beyond the species level was nearly impossible due to considerable variability in length, sequence composition, and insertions/deletions (data not shown). The majority of the remaining spacer regions were short, with a few exceptions (*e.g.*, less than 10 bp).

**Figure 4:**
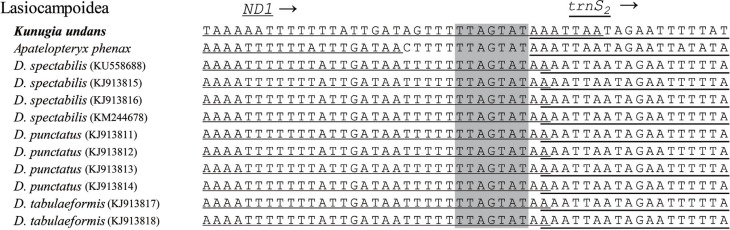
Alignment of the internal spacer sequence located between *ND1* and *trnS*_*2*_ of Lasiocampoidea, including *Kunugia undans*. The shaded nucleotides indicate the conserved heptanucleotide (TTAGTAT) region. Underlined nucleotides indicate the adjacent partial sequences of *ND1* and *trnS*_*2*_. Arrows indicate the transcriptional direction.

In previous lepidopteran mitogenomic studies, other spacer sequences at the *trnS*_*2*_ and *ND1* junction were consistently reported in lepidopteran lineages (Cameron and Whiting, 2008; Kim *et al.*, 2010; Yang *et al.*, 2013; Kim *et al.*, 2014; Park *et al.*, 2016). The important feature of this spacer is the presence of a short-length TTAGTAT motif within the spacer sequence, which is thought to be a possible binding site for the transcription termination peptide of mtDNA (mtTERM). This characterization is based on the fact that the spacer is located after the final major-strand coded *CytB* (Taanman, 1999; Cameron and Whiting, 2008). Regarding *K. undans*, there is a 7-bp overlap at the *ND1* and *trnS*_*2*_ junction, but *K. undans* clearly possesses the same sequence motif (Figure 4). All other lasiocampid species, with the exception of *A. phenax*, have a 1-bp gene overlap in this region, but they also contain the 7-bp motif at the *ND1* and *trnS*_*2*_ junction. On the other hand, *A. phenax* has an intergenic spacer sequence at 12 bp, which includes the 7-bp motif. In other macroheteroceran species, the 7-bp motif is found in nearly all species without modification, with the exception of one Noctuoidea species, which has ATAGTAT instead of TTAGTAT. In Macroheterocera, the 7-bp motif is nearly always located at the spacer instead of the coding region at the *ND1* and *trnS*_*2*_ junction (Figure S5). Thus, the spacing pattern of Lasiocampoidea differs from that of other macroheteroceran superfamilies in this region, so mRNA expression data would be required to clarify the extension of *ND1* at the *ND1* and *trnS*_*2*_ junction.

In summary, in addition to the typical set of genes, the 15,570-bp complete mitogenome sequence of *K. undans* has an extra *trnR*. The presence of the additional tRNA is unique in Macroheterocera, including Lasiocampoidea. The A+T-rich region of *K. undans* possesses a few conserved sequences, which were previously reported in other Macroheterocera (including Lasiocampoidea). Moreover, the intergenic spacing pattern and size for *K. undans* are largely consistent with those of other Macroheterocera (including Lasiocampoidea), but instead of an intergenic spacer, Lasiocampoidea (including *K. undans*) exhibit an overlap at the *trnS*_*2*_ and *ND1* junction.

## Supplementary material

The following online material is available for this article:

Table S1 - List of primers used to amplify and sequence the *Kunugia undans* mitogenome.

Table S2 - Characteristics of Macroheterocera mitogenomes.

Table S3 - Genomic summary of Lasiocampoidea.

Table S4 - Frequency of the four most frequently used codons in Macroheterocera.

Figure S1 - Predicted secondary cloverleaf structures for the 23 tRNA genes of *Kunugia undans*, with the duplicated *trnR* (A and B).

Figure S2 - Alignment of the initiation context of the Macroheterocera *COI*.

Figure S3 - Schematic illustration of the A+T-rich region of Macroheterocera.

Figure S4 - Alignment of the spacer sequence (located between *trnQ* and *ND2*) and neighboring partial Macroheterocera *ND2*.

Figure S5 - Alignment of the internal spacer sequence located between *ND1* and *trnS*_*2*_ of Macroheterocera.
