# Supplementary material for: Complete mitochondrial genome of the lappet moth, Kunugia undans (Lepidoptera: Lasiocampidae): genomic comparisons among macroheteroceran superfamilies
Source: Genet Mol Biol. 2017 Jul 31;40(3):717–23. doi: 10.1590/1678-4685-GMB-2016-0298 (PMC5596373; doi:10.1590/1678-4685-GMB-2016-0298)
Supplement: Supplementary file 1 [file 1415-4757-gmb-1678-4685-GMB-2016-0298-Suppl01.pdf]

Genetics and Molecular Biology, 40, 3 (2017)

Supplementary Material to "Complete mitochondrial genome of the lappet moth, Kunugia undans (Lepidoptera: Lasiocampidae): genomic comparisons among macroheteroceran superfamilies"

Table S1 - List of primers used to amplify and sequence the *Kunugia undans* mitogenome.

Fragment name	Primer name	Direction[a]	Sequence (5'- 3')	Nucleotide position[b]	Mismatch[c]
Long fragments					
LF1	Lep-COI-F2	F	ATAGTTATACCTATTATAATTGG	1695-1717	–
〃	Lep-ND4-R2	R	GCTCATGTTGAAGCTCCTGT	9045-9064	3
LF2	Lep-ND5-F1	F	CTAAAAGGAATTTGAGCTCT	7671-7690	1
〃	Lep-lrRNA-R2	R	GTATCTTGTGTATCAGAGTTTA	14215-14236	–
LF3	Lep-lrRNA-F1	F	TGTAAGATTTTAATGATCGAACAGAT	13187-13212	–
〃	Lep-COI-R1	R	CTTCAGGATGACCAAAAAATC	2197-2217	2
Short fragments					
SF1	LF03-S05-F1	F	AAGCTTTTGGGYTCATACC	20-38	0
〃	LF03-S05-R2	R	CAWCCTAAATTATTAATWGAWGA	794-816	3
SF2	LF03-S06-F1	F	TCWTCHWTATTAATAAAAATAGG	557+579	1
〃	LF03-S06-R2	R	GCGATAAATTGTAAATTTAT	1436-1455	0
SF3	LF03-S07-F2	F	CTTAAAATTTGCAATTTTATATC	1362-1384	1
〃	Lep-COI-R1	R	CTTCAGGATGACCAAAAAATC	2197-2217	2
SF4	LF01-S01-F1	F	GGWATTTCWTCAATTTTAGG	1950-1969	3
〃	LF01-S01-R2	R	GTCGAGGTATTCCTGCTA	2815-2832	0
SF5	LF01-S02-F2	F	ACWGTAGGAGGATTAACAGG	2802-2821?	7
〃	LF01-S02-R2	R	GTTCAAATTAATTCAATTATTTG	3299-3321	0
SF6	LF01-S03-F2	F	TAGAAATGGCAACWTGATC	3120-3138	1
〃	LF01-S03-R1	R	CTTGCTTTCAGTCATCTAAT	3808-3827	0
SF7	LF01-S04-F1	F	CAGGTCGWTTAAATCAAAC	3651-3669	2
〃	LF01-S04-R1	R	CTAGTTCTDGTAAAAATATA	4376-4395	1
SF8	LF01-S05-F2	F	TTTTATTTAATAATTTTTTAGG	4344-4365	4
〃	LF01-S05-R1	R	CTCGTCATCATTGATATAT	4949-4967	2
SF9	LF01-S06-F2	F	GTWGATTATAGHCCWTGACC	4820-4839	0
〃	LF01-S06-R2	R	GATTGGAAGTCAAATATACT	5595-5614	0
SF10	LF01-S07-F1	F	AGCATATGAATATWTWGAAGC	5320-5340	2
〃	LF01-S07-R1	R	CAATTTTATCATTAACAGTGA	6268-6288	0
SF11	LF01-S08-F2	F	GAAATCAAAATATATTAAATTG	5967-5988	1
〃	LF01-S08-R2	R	CCTTATATAATTTATTTACC	6918-6937	1
SF12	LF01-S09-F1	F	AWAHTTCTCTTCAACCYAWATC	6682-6703	1
	SF12-HN-F[d]	F	TATCTTCAATATTACACTCTG	6499-6519	2
〃	LF01-S09-R2	R	GCTTTATCWACTTTAAGWCA	7437-7456	0

Fragment name	Primer name	Direction[a]	Sequence (5'- 3')	Nucleotide position[b]	Mismatch[c]
SF13	LF01-S10-F1	F	TCYTTWGAATAAAAYCCAG	7185-7203	1
〃	LF01-S10-R1	R	GATGGDTTAGGDTTAGTTTCTT	7906-7927	0
SF14	LF01-S11-F1	F	AAAAAATATAATTTCAWCTHCC	7765-7786	0
〃	LF01-S11-R1	R	CATTGATTWCCTTTAAATAT	8385-8404	1
SF15	LF01-S12-F1	F	ATATTTTTGAYHCCACAAATC	8298-8318	0
〃	LF01-S12-R1	R	CAGGTTCAATAATTTTAGC	9024-9042	2
SF16	LF02-S01-F2	F	TGAGCWACWGAAGAATAAGC	8841-8860	0
〃	LF02-S01-R1	R	GGTTTAATTTTATTAAGAATTTG	9483-9505	3
SF17	LF02-S02-F1	F	ATATTAAAGTAGGAATTAAWC	9307-9327	0
〃	LF02-S02-R2	R	TAATTTTGGAGATTATWGAT	10072-10091	0
SF18	LF02-S03-F1	F	CCTAAAGCHCCYTCACAAAC	9758-9777	2
〃	LF02-S03-R1	R	GTAATTTTTACWACTGCAATTA	10596-10617	0
SF19	LF02-S04-F1	F	TTAAWACATATTGATTTTCTTA	10257-10278	1
	SF19-HN-F[d]		AATTTTTATAACAAAAATGAAATAT G	9635-9653	5
〃	LF02-S04-R1	R	GATATTTGTCCYCAAGGTA	11071-11089	5
SF20	LF02-S05-F2	F	TATHTHCATATTGGACGAGG	10956-10975	1
〃	LF02-S05-R2	R	CCAATTCAWGTTAATAAAAT	11667-11686	1
SF21	LF02-S06-F2	F	ACHCCHRTTCATATTCAACC	11460-11479	2
〃	LF02-S06-R1	R	GAGAATTAGTTTCAGGRTTTA	12171-12191	1
SF22	LF02-S07-F1	F	AACGAGGTAAWGTHCCHCG	11988-12006	0
〃	LF02-S07-R2	R	CTGAGTTCAAACCGGTGTRA	13163-13182	0
SF23	LF02-S08-F1	F	TCTAATAAAGTTAAAAAAGC	12734-12753	3
〃	LF02-S08-R2	R	CACTTGTTTATCAAAAACATGTC	13703-13725	0
SF24	LF03-S02-F1	F	ATTATGCTACCTTTGTACAGTC	13617-13638	0
〃	LF03-S02-R1	R	GTATTTCATTTACATTGAAAAGA	14430-14452	1
SF25	LF03-S03-F3	F	CTCTGATACACAAGATAC	14219-14236	0
〃	LF03-S03-R3	R	CCAGCAGTTGCGGTTAAAC	15064-15082	0
SF26	LF03-S04-F1	F	AATAGGTGATCTAATCCTAG	14909-14928	2
	SF26-CB-F[e]	F	TATAACCGCAATTGCTGGC	15065-15083	2
〃	LF03-S04-R2	R	GRTATAGTTTAATTCTATAAA	143-163	1

[a]F and R: forward and reverse transcriptional directions.

[b, c]Nucleotide positions and mismatches with respect to the *Kunugia undans* mitogenome.
