# Supplementary material for: Complete mitochondrial genome of the lappet moth, Kunugia undans (Lepidoptera: Lasiocampidae): genomic comparisons among macroheteroceran superfamilies
Source: Genet Mol Biol. 2017 Jul 31;40(3):717–23. doi: 10.1590/1678-4685-GMB-2016-0298 (PMC5596373; doi:10.1590/1678-4685-GMB-2016-0298)
Supplement: Supplementary file 2 [file 1415-4757-gmb-1678-4685-GMB-2016-0298-Suppl02.pdf]

Supplementary Material to "Complete mitochondrial genome of the lappet moth, Kunugia undans (Lepidoptera: Lasiocampidae): genomic comparisons among macroheteroceran superfamilies"

Table S2 - Characteristics of Macroheterocera mitogenomes.

Taxon	Size (bp)	A/T content (%)	PCG		srRNA		lrRNA		tRNA		A+T-rich region		GenBank accession no.
			No. codons[a]	AT%	Size (bp)	AT%	Size (bp)	AT%	Size (bp)	AT%	Size (bp)	AT%	
**Bombycoidea**													
Saturniidae													
Saturniinae													
Attacini													
Samia cynthia ricini	15,384	79.78	3,732	78.38	779	83.83	1,358	84.02	1,463	80.59	361	90.86	JN215366
Attacus atlas	15,282	79.30	3,727	77.68	777	83.14	1,368	84.80	1,436	81.20	357	90.48	KF006326
Saturniini													
Antheraea yamamai	15,338	80.29	3,729	78.89	776	84.41	1,380	83.99	1,473	81.33	334	89.52	EU726630
Saturnia boisduvalii	15,360	80.62	3,734	79.10	774	84.11	1,391	84.76	1,478	81.87	330	91.52	EF622227
Eriogyna pyretorum	15,327	80.82	3,731	79.35	778	84.45	1,338	84.60	1,477	81.86	358	92.18	FJ685653
Actias aliena	15,243	78.62	3,729	76.85	777	84.30	1,363	83.13	1,462	80.78	328	91.77	KF927042
Bombycidae													
Bombycinae													
Bombyx huttoni	15,638	81.80	3,719	80.04	777	86.74	1,399	85.28	1,476	81.57	512	95.12	KP216766
Rondotia menciana	15,364	82.14	3,730	77.04	782	84.40	1,365	83.37	1,485	81.01	360	91.11	KJ647172
Sphingidae													
Sphinginae													
Manduca sexta	15,516	81.79	3,718	80.24	777	85.71	1,391	85.26	1,484	81.74	324	95.37	EU286785
Sphinx morio	15,229	81.17	3,716	79.78	773	85.25	1,379	84.55	1,463	81.61	316	92.72	KC470083
**Geometroidea**													
Geometridae													
Ennominae													
Phthonandria atrilineata	15,499	81.02	3,724	79.05	803	86.30	1,400	85.71	1,476	81.44	457	98.25	EU569764
Biston panterinaria	15,517	79.55	3,728	77.33	787	84.75	1,474	85.55	1,477	81.65	349	93.12	JX406146
Apocheima cinerarium	15,722	80.85	3,732	78.77	789	85.68	1,390	84.82	1,483	81.46	625	95.84	KF836545
Celenna sp.	15,403	80.75	3,726	79.03	785	85.22	1,370	85.69	1,485	81.82	331	92.15	KM244697
Jankowskia athleta	15,534	79.53	3,728	77.66	785	84.20	1,386	83.62	1,489	81.26	675	91.41	KR822683
Larentiinae													

Taxon	Size (bp)	A/T content (%)	PCG		srRNA		lrRNA		tRNA		A+T-rich region		GenBank accession no.
			No. codons[a]	AT%	Size (bp)	AT%	Size (bp)	AT%	Size (bp)	AT%	Size (bp)	AT%	
Dysstroma truncata	15,828	79.95	3,730[b]	77.77	748	85.16	1,381	83.49	1,475	81.29	868	93.55	KJ508061
Operophtera brumata	15,748	79.97	3,716	78.00	771	85.08	1,306	83.69	1,466	80.90	801	91.26	KP027400
**Noctuoidea**													
Notodontidae													
Thaumetopoeinae													
Ochrogaster lunifer	15,593	77.84	3,745	75.67	806	83.25	1,351	81.50	1,502	81.29	319	93.42	AM946601
Phalerinae													
Phalera flavescens	15,659	80.87	3,728	78.91	830	85.78	1,368	85.09	1,485	82.22	541	91.87	JF440342
Erebidae													
Lymantriinae													
Lymantria dispar	15,569	79.88	3,735	77.78	799	85.23	1,351	84.23	1,469	81.01	435	96.09	FJ617240
Gynaephora menyuanensis	15,770	81.48	3,731	79.72	891	85.52	1,420	84.23	1,501	83.08	449	93.32	KC185412
Lachana alpherakii	15,755	81.44	3,729	79.62	850	84.94	1,484	85.31	1,502	83.02	449	93.10	KJ957168
Euproctis pseudoconspersa	15,461	79.93	3,718	77.93	812	84.73	1,413	84.64	1,466	81.58	388	93.81	KJ716847
Arctiinae													
Hyphantria cunea	15,481	80.39	3,724	78.53	808	84.53	1,426	84.99	1,474	81.75	357	94.96	GU592049
Callimorpha dominula	15,496	81.02	3,728	80.16	782	84.53	1,372	84.55	1,462	82.08	486	75.10	KP973953
Vamuna virilis	15,417	80.39	3,724	78.52	778	85.09	1,394	84.65	1,456	81.52	362	95.03	KJ364659
Lemyra melli	15,418	78.67	3,723	76.60	806	84.74	1,427	83.88	1,486	80.82	338	94.38	KP307017
Cyana sp.	15,494	81.20	3,732	79.47	757	84.94	1,436	84.54	1,475	81.69	379	95.25	KM244679
Nyctemera arctata	15,432	80.05	3,725	79.28	800[b]	78.75	1,358[b]	81.30	1,445	81.11	400	94.00	KM244681
Syntominae													
Amata formosae	15,463	79.49	3,730	77.74	792	84.97	1,371	83.08	1,467	80.71	482	92.74	KC513737
Aganainae													
Asota plana lacteata	15,416	80.35	3,726	78.57	787	85.01	1,398	84.62	1,462	80.98	328	94.51	KJ173908
Noctuidae													
Acronictinae													
Acronicta psi	15,350	79.09	3,727[b]	77.21	745	84.56	1,378	82.95	1,472	81.52	388	92.53	KJ508060
Heliothinae													
Helicoverpa armigera	15,347	80.97	3,724	79.38	794	85.89	1,395	84.73	1,474	81.82	328	95.12	GU188273
Heliothis subflexa	15,323	80.73	3,722	79.16	776	85.31	1,358	84.09	1,460	81.44	323	95.67	KT598688
Amphipyrinae													
Sesamia inferens	15,413	80.24	3,733	78.56	784	85.33	1,385	83.39	1,478	81.53	311	95.82	JN039362
Spodoptera litura	15,388	80.98	3,724	79.46	811	84.71	1,397	84.68	1,477	81.92	330	93.94	JQ647918
Plusiinae													
Ctenoplusia agnata	15,261	81.10	3,735	79.85	784	85.46	1,328	84.19	1,477	81.45	334	93.41	KC414791
Noctuinae													

Taxon	Size (bp)	A/T content (%)	PCG		srRNA		lrRNA		tRNA		A+T-rich region		GenBank accession no.
			No. codons[a]	AT%	Size (bp)	AT%	Size (bp)	AT%	Size (bp)	AT%	Size (bp)	AT%	
Agrotis ipsilon	15,377	81.25	3,726	79.75	784	85.46	1,378	84.98	1,477	81.65	332	94.88	KF163965
Noctua pronuba	15,315	81.06	3,715[b]	79.61	751	85.89	1,379	84.63	1,469	81.21	365	93.42	KJ508057
Striacosta albicosta	15,553	79.33	3,725	77.40	771	85.08	1,376	83.36	1,486	81.02	385	92.99	KM488268
Hadeninae													
Mythimna separata	15,332	80.99	3,726	79.51	784	85.71	1,358	84.24	1,486	81.02	374	94.39	KF730242
Euteliinae													
Eutelia adulatricoides	15,360	80.87	3,726	79.34	783	84.67	1,361	84.50	1,476	81.84	341	92.67	KJ185131
Catocalinae													
Catocala sp.	15,671	81.12	3,730	79.45	790	85.70	1,471	84.91	1,475	81.76	425	90.59	KJ432280
Nolidae													
Nolinae													
Risoba prominens	15,343	81.07	3,727	79.58	816	85.54	1,387	85.51	1,465	81.91	342	93.57	KJ396197
Chloephorinae													
Gabala argentata	15,337	81.69	3,724	80.27	785	85.99	1,380	85.58	1,469	81.55	340	95.29	KJ410747
**Drepanoidea**													
Drepanidae													
Drepana arcuata	15,302	81.13	3,712[b]	79.50	752	84.84	1,360	84.93	1,475	81.29	380	94.21	KJ508053
Doidae													
Doa sp.	15,228	80.44	3,710[b]	78.84	744	85.08	1,331	83.70	1,472	81.39	454	95.37	KJ508058
**Mimallonoidea**													
Mimallonidae													
Lacosoma valva	16,108	80.87	3,728[b]	78.34	746	85.39	1,420	85.77	1,472	80.98	538	96.47	KJ508050

[a]Stop codons were excluded from the total codon count.
