# Supplementary material for: Complete mitochondrial genome of the lappet moth, Kunugia undans (Lepidoptera: Lasiocampidae): genomic comparisons among macroheteroceran superfamilies
Source: Genet Mol Biol. 2017 Jul 31;40(3):717–23. doi: 10.1590/1678-4685-GMB-2016-0298 (PMC5596373; doi:10.1590/1678-4685-GMB-2016-0298)
Supplement: Supplementary file 3 [file 1415-4757-gmb-1678-4685-GMB-2016-0298-Suppl03.pdf]

Genetics and Molecular Biology, 40, 3 (2017)

**Supplementary Material to "Complete mitochondrial genome of the lappet moth, Kunugia undans (Lepidoptera: Lasiocampidae): genomic comparisons among macroheteroceran superfamilies"**

**Table S3 -** Genomic summary of Lasiocampoidea.

| Gene | Anticodon | Start codon | Stop codon | ***Kunugia undans*** | *Apatelopteryx phenax* | *D. spectabilis* (KU558688) | *D. spectabilis* (KJ913815) | *D. spectabilis* (KJ913816) |
|---|---|---|---|---|---|---|---|---|
| *trnM* | CAT | | | 1-68(68) | 1-68(68) | 1-67(67) | 1-67(67) | 1-67(67) |
| *trnI* | GAT | | | 72-135(64) | 77-141(65) | 71-134(64) | 71-134(64) | 71-134(64) |
| *trnQ* | TTG | | | 136-205(70) | 139-207(69) | 132-200(69) | 132-200(69) | 132-200(69) |
| *ND2* | | ATT[a]/ATG[b] | TAA | 263-1276(1014)[a] | 247-1278(1032)[b] | 259-1266(1008)[a] | 259-1266(1008)[a] | 259-1266(1008)[a] |
| *trnW* | TCA | | | 1275-1344(70) | 1277-1347(71) | 1265-1333(69) | 1265-1333(69) | 1265-1333(69) |
| *trnC* | GCA | | | 1337-1402(66) | 1340-1407(68) | 1326-1391(66) | 1326-1391(66) | 1326-1391(66) |
| *trnY* | GTA | | | 1412-1479(68) | 1417-1483(66) | 1392-1459(68) | 1392-1459(68) | 1392-1459(68) |
| *COI* | | CGA | T-tRNA | 1500-3057(1558) | 1502-3032(1531) | 1485-3015(1531) | 1485-3015(1531) | 1485-3015(1531) |
| *trnL*$_2$ | TAA | | | 3058-3125(67) | 3033-3100(68) | 3016-3082(71) | 3016-3082(71) | 3016-3082(71) |
| *COII* | | ATA | T-tRNA | 3125-3806(682) | 3101-3782(682) | 3083-3764(682) | 3083-3764(682) | 3083-3764(682) |
| *trnK* | CTT | | | 3807-3877(71) | 3783-3853(71) | 3765-3835(71) | 3765-3835(71) | 3765-3835(71) |
| *trnD* | GTC | | | 3879-3947(69) | 3870-3936(67) | 3839-3906(68) | 3839-3906(68) | 3839-3906(68) |
| *ATP8* | | ATC[a]/ATT[b] | TAA | 3948-4109(162)[a] | 3937-4110(174)[a] | 3907-4065(159)[a] | 3907-4065(159)[a] | 3907-4065(159)[a] |
| *ATP6* | | ATG | TAA | 4103-4780(678) | 4104-4781(678) | 4059-4736(678) | 4059-4736(678) | 4059-4736(678) |
| *COIII* | | ATG | TAA | 4787-5575(790) | 4795-5583(789) | 4748-5536(789) | 4748-5536(789) | 4748-5536(789) |
| *trnG* | TCC | | | 5578-5644(67) | 5586-5651(66) | 5539-5604(66) | 5539-5604(66) | 5539-5604(66) |
| *ND3* | | ATC[a]/ATT[b]/ATG[c]/ATA[d] | TAA | 5645-5998(354)[a] | 5655-6005(351)[b] | 5605-5958(354)[a] | 5605-5958(354)[a] | 5605-5958(354)[a] |
| *trnA* | TGC | | | 6003-6070(68) | 6044-6111(68) | 5958-6024(67) | 5958-6024(67) | 5958-6024(67) |
| *trnR* (A) | TCG | | | 6084-6147(64) | 6125-6191(67) | 6045-6108(64) | 6045-6108(64) | 6045-6108(64) |
| *trnR* (B) | TCG | | | 6175-6241(67) | | | | |
| *trnN* | GTT | | | 6242-6308(67) | 6209-6275(67) | 6113-6178(66) | 6113-6178(66) | 6113-6178(66) |
| *trnS*$_1$ | GCT | | | 6308-6375(68) | 6297-6362(66) | 6197-6264(68) | 6197-6264(68) | 6197-6264(68) |
| *trnE* | TTC | | | 6376-6440(65) | 6363-6428(66) | 6264-6329(66) | 6264-6328(65) | 6264-6328(65) |
| *trnF* | GAA | | | 6471-6537(67) | 6467-6535(69) | 6338-6403(66) | 6337-6402(66) | 6337-6402(66) |
| *ND5* | | ATT | T-tRNA[a]/TAA[b] | 6538-8275(1738)[a] | 6535-8255(1721)[a] | 6407-8149(1743)[b] | 6406-8148(1743)[b] | 6406-8148(1743)[b] |

| Gene | Anti codon | Start codon | Stop codon | ***Kunugia undans*** | *Apatelopteryx phenax* | *D. spectabilis* (KU558688) | *D. spectabilis* (KJ913815) | *D. spectabilis* (KJ913816) |
|---|---|---|---|---|---|---|---|---|
| *trnH* | GTG | | | 8276-8343(68) | 8271-8336(66) | 8150-8217(68) | 8149-8216(68) | 8149-8216(68) |
| *ND4* | | ATG[a]/ATA[b] | TAG[1]/T-tRNA[2] | 8348-9682(1335)[a, 1] | 8336-9690(1355)[b,2] | 8218-9556(1339)[a, 2] | 8217-9555(1339)[a, 2] | 8217-9555(1339)[a, 2] |
| *ND4L* | | ATG[a]/ATT[b] | TAG[1]/TAA[2] | 9688-9981(294)[a, 1] | 9687-9980(294)[a, 2] | 9580-9867(288)[b, 2] | 9579-9872(294)[a, 2] | 9579-9872(294)[a, 2] |
| *trnT* | TGT | | | 9986-10050(65) | 9985-10049(65) | 9881-9944(64) | 9880-9943(64) | 9880-9943(64) |
| *trnP* | TGG | | | 10051-10115(65) | 10050-10114(65) | 9945-10009(65) | 9944-10008(65) | 9944-10008(65) |
| *ND6* | | ATA | TAA | 10124-10654(531) | 10123-10662(540) | 10018-10548(531) | 10017-10547(531) | 10017-10547(531) |
| *CytB* | | ATG[a]/ATA[b] | TAA | 10662-11807(1146)[a] | 10665-11819(1155)[b] | 10553-11701(1149)[a] | 10552-11700(1149)[a] | 10552-11700(1149)[a] |
| *trnS*$_2$ | TGA | | | 11809-11875(67) | 11836-11903(68) | 11705-11770(66) | 11704-11769(66) | 11704-11769(66) |
| *ND1* | | ATG | TAA | 11869-12825(957) | 11916-12854(939) | 11770-12723(954) | 11769-12722(954) | 11769-12722(954) |
| *trnL*$_1$ | TAG | | | 12827-12892(66) | 12856-12921(66) | 12725-12792(68) | 12724-12791(68) | 12724-12791(68) |
| *lrRNA* | | | | 12893-14406(1513) | 12922-14267(1346) | 12793-14246(1454) | 12792-14245(1454) | 12792-14245(1454) |
| *trnV* | TAC | | | 14407-14471(65) | 14268-14347(80) | 14247-14312(66) | 14246-14311(66) | 14246-14311(66) |
| *srRNA* | | | | 14472-15253(782) | 14348-15094(747) | 14313-15089(777) | 14312-15092(780) | 14312-15090(779) |
| A+T–rich region | | | | 15254-15570(317) | 15095-15552(458) | 15090-15409(320) | 15093-15412(319) | 15091-15410(319) |

| *D. spectabilis* (KM244678) | *D. punctatus* (KJ913811) | *D. punctatus* (KJ913812) | *D. punctatus* (KJ913813) | *D. punctatus* (KJ913814) | *D. tabulaeformis* (KJ913817) | *D. tabulaeformis* (KJ913818) |
|---|---|---|---|---|---|---|
| 445-511(67) | 1-67(68) | 1-67(68) | 1-67(68) | 1-67(68) | 1-67(68) | 1-67(68) |
| 515-578(64) | 71-134(64) | 71-134(64) | 71-134(64) | 71-134(64) | 71-134(64) | 71-134(64) |
| 576-644(69) | 132-200(69) | 132-200(69) | 132-200(69) | 132-200(69) | 132-200(69) | 132-200(69) |
| 703-1710(1008)[a] | 259-1266(1008)[a] | 259-1266(1008)[a] | 259-1266(1008)[a] | 259-1266(1008)[a] | 259-1266(1008)[a] | 259-1266(1008)[a] |
| 1709-1777(69) | 1265-1333(69) | 1265-1333(69) | 1265-1333(69) | 1265-1333(69) | 1265-1333(69) | 1265-1333(69) |
| 1770-1835(66) | 1326-1391(66) | 1326-1391(66) | 1326-1391(66) | 1326-1391(66) | 1326-1391(66) | 1326-1391(66) |
| 1836-1903(68) | 1392-1459(68) | 1392-1459(68) | 1392-1459(68) | 1392-1459(68) | 1392-1459(68) | 1392-1459(68) |
| 1929-3459(1531) | 1494-3024(1531) | 1494-3024(1531) | 1487-3017(1531) | 1494-3024(1531) | 1494-3024(1531) | 1494-3024(1531) |
| 3460-3526(67) | 3025-3091(67) | 3025-3091(67) | 3018-3084(67) | 3025-3091(67) | 3025-3091(67) | 3025-3091(67) |
| 3527-4208(682) | 3092-3773(682) | 3092-3773(682) | 3085-3766(682) | 3092-3773(682) | 3092-3773(682) | 3092-3773(682) |
| 4209-4279(71) | 3774-3844(71) | 3774-3844(71) | 3767-3837(71) | 3774-3844(71) | 3774-3844(71) | 3774-3844(71) |
| 4283-4350(68) | 3848-3915(68) | 3848-3915(68) | 3841-3908(68) | 3848-3915(68) | 3848-3915(68) | 3848-3915(68) |

| *D. spectabilis* (KM244678) | *D. punctatus* (KJ913811) | *D. punctatus* (KJ913812) | *D. punctatus* (KJ913813) | *D. punctatus* (KJ913814) | *D. tabulaeformis* (KJ913817) | *D. tabulaeformis* (KJ913818) |
|---|---|---|---|---|---|---|
| 4351-4509(159)[a] | 3916-4077(162)[b] | 3916-4077(162)[b] | 3909-4070(162)[b] | 3916-4077(162)[b] | 3916-4074(159)[b] | 3916-4074(159)[a] |
| 4503-5180(678) | 4071-4748(678) | 4071-4748(678) | 4064-4741(678) | 4071-4748(678) | 4068-4745(678) | 4068-4745(678) |
| 5192-5980(789) | 4764-5552(789) | 4764-5552(789) | 4757-5545(789) | 4763-5551(789) | 4761-5549(789) | 4761-5549(789) |
| 5983-6048(66) | 5555-5620(66) | 5555-5620(66) | 5548-5613(66) | 5554-5619(66) | 5552-5617(66) | 5552-5617(66) |
| 6049-6402(354)[a] | 5621-5974(354)[c] | 5621-5974(354)[c] | 5614-5967(354)[c] | 5620-5973(354)[d] | 5618-5971(354)[c] | 5618-5971(354)[b] |
| 6402-6468(67) | 5974-6040(67) | 5974-6040(67) | 5967-6033(67) | 5973-6039(67) | 5971-6037(67) | 5971-6037(67) |
| 6489-6552(64) | 6056-6119(63) | 6056-6119(63) | 6049-6112(63) | 6055-6118(63) | 6053-6116(64) | 6053-6116(64) |
| 6557-6622(66) | 6124-6189(66) | 6124-6189(66) | 6117-6182(66) | 6123-6188(66) | 6121-6186(66) | 6121-6186(66) |
| 6642-6707(66) | 6201-6268(68) | 6201-6268(68) | 6194-6261(68) | 6202-6269(68) | 6198-6265(68) | 6198-6265(68) |
| 6708-6772(65) | 6268-6333(66) | 6268-6333(66) | 6261-6326(66) | 6269-6333(65) | 6265-6329(65) | 6265-6329(65) |
| 6781-6846(66) | 6338-6403(66) | 6337-6402(66) | 6331-6396(66) | 6338-6403(66) | 6334-6399(66) | 6334-6399(66) |
| 6850-8592(1743)[b] | 6406-8148(1743)[b] | 6405-8147(1743)[b] | 6399-8141(1743)[b] | 6407-8149(1743)[b] | 6402-8144(1743)[b] | 6402-8144(1743)[b] |
| 8593-8660(68) | 8149-8216(68) | 8148-8215(68) | 8142-8209(68) | 8150-8217(68) | 8145-8212(68) | 8145-8212(68) |
| 8661-9999(1339)[a,2] | 8217-9555(1339)[a,2] | 8216-9554(1339)[a,2] | 8210-9548(1339)[a,2] | 8218-9556(1339)[a,2] | 8213-9551(1339)[a,2] | 8213-9551(1339)[a,2] |
| 10023-10310(288)[b,2] | 9580-9873(294)[a,2] | 9579-9872(294)[a,2] | 9573-9866(294)[a,2] | 9576-9869(294)[a,2] | 9576-9869(294)[a,2] | 9576-9869(294)[a,2] |
| 10324-10387(64) | 9881-9944(64) | 9880-9943(64) | 9874-9937(64) | 9877-9941(64) | 9877-9940(64) | 9877-9940(64) |
| 10388-10452(65) | 9945-10009(65) | 9944-10008(65) | 9938-10002(65) | 9942-10006(65) | 9941-10005(65) | 9941-10005(65) |
| 10413-10991(578) | 10018-10548(531) | 10017-10547(531) | 10011-10541(531) | 10015-10545(531) | 10014-10544(531) | 10014-10544(531) |
| 10996-12144(1148)[a] | 10553-11701(1149)[a] | 10552-11700(1149)[a] | 10546-11694(1149)[a] | 10550-11698(1149)[a] | 10549-11697(1149)[a] | 10549-11697(1149)[a] |
| 12148-12213(66) | 11705-11771(67) | 11704-11770(67) | 11698-11764(67) | 11702-11768(67) | 11701-11767(67) | 11701-11767(67) |
| 12213-13166(954) | 11771-12724(954) | 11770-12723(954) | 11764-12717(954) | 11768-12721(954) | 11767-12720(954) | 11767-12720(954) |
| 13168-13235(68) | 12726-12793(68) | 12725-12792(68) | 12719-12786(68) | 12723-12790(68) | 12722-12789(68) | 12722-12789(68) |
| 13337-14693(1356) | 12794-14255(1462) | 12793-14254(1462) | 12787-14247(1461) | 12791-14242(1462) | 12790-14248(1459) | 12790-14245(1456) |
| 14690-14755(66) | 14256-14320(65) | 14255-14319(65) | 14248-14312(65) | 14243-14307(65) | 14249-14313(65) | 14246-14310(65) |
| 14763-15390(628) | 14321-15099(779) | 14320-15098(779) | 14313-15091(779) | 14308-15087(780) | 14314-15091(778) | 14311-15089(779) |
| 15391-15411(20) | 15100-15419(320) | 15099-15418(320) | 15092-15411(320) | 15088-15407(320) | 15092-15411(320) | 15090-15409(320) |
| 1-444(444) | | | | | | |

Superscripts indicate identical start and stop codons among the lasiocampid species. Values in parentheses indicate gene size (bp).
