# Supplementary material for: Complete mitochondrial genome of the lappet moth, Kunugia undans (Lepidoptera: Lasiocampidae): genomic comparisons among macroheteroceran superfamilies
Source: Genet Mol Biol. 2017 Jul 31;40(3):717–23. doi: 10.1590/1678-4685-GMB-2016-0298 (PMC5596373; doi:10.1590/1678-4685-GMB-2016-0298)
Supplement: Supplementary file 4 [file 1415-4757-gmb-1678-4685-GMB-2016-0298-Suppl05.pdf]

Genetics and Molecular Biology, 40, 3 (2017)

Supplementary Material to "Complete mitochondrial genome of the lappet moth, Kunugia undans (Lepidoptera: Lasiocampidae): genomic comparisons among macroheteroceran superfamilies"

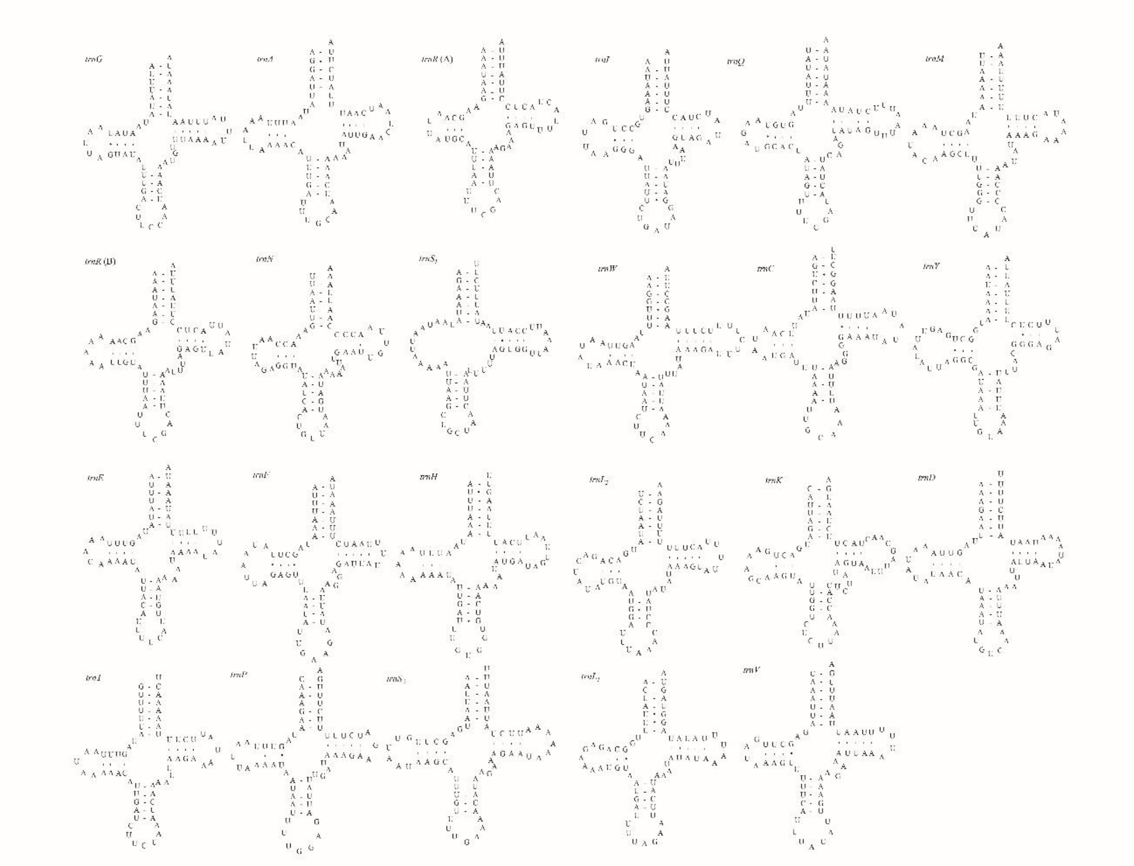

Figure S1 - Predicted secondary cloverleaf structures for the 23 tRNA genes of *Kunugia undans*, with the duplicated *trnR* (A and B). Dashes (-) indicate Watson-Crick base-pairing, and centered dots (•) indicate G-U base pairing.
