# Supplementary material for: Complete mitochondrial genome of the lappet moth, Kunugia undans (Lepidoptera: Lasiocampidae): genomic comparisons among macroheteroceran superfamilies
Source: Genet Mol Biol. 2017 Jul 31;40(3):717–23. doi: 10.1590/1678-4685-GMB-2016-0298 (PMC5596373; doi:10.1590/1678-4685-GMB-2016-0298)
Supplement: Supplementary file 5 [file 1415-4757-gmb-1678-4685-GMB-2016-0298-Suppl06.pdf]

Supplementary Material to "Complete mitochondrial genome of the lappet moth, Kunugia undans (Lepidoptera: Lasiocampidae): genomic comparisons among macroheterocean superfamilies"

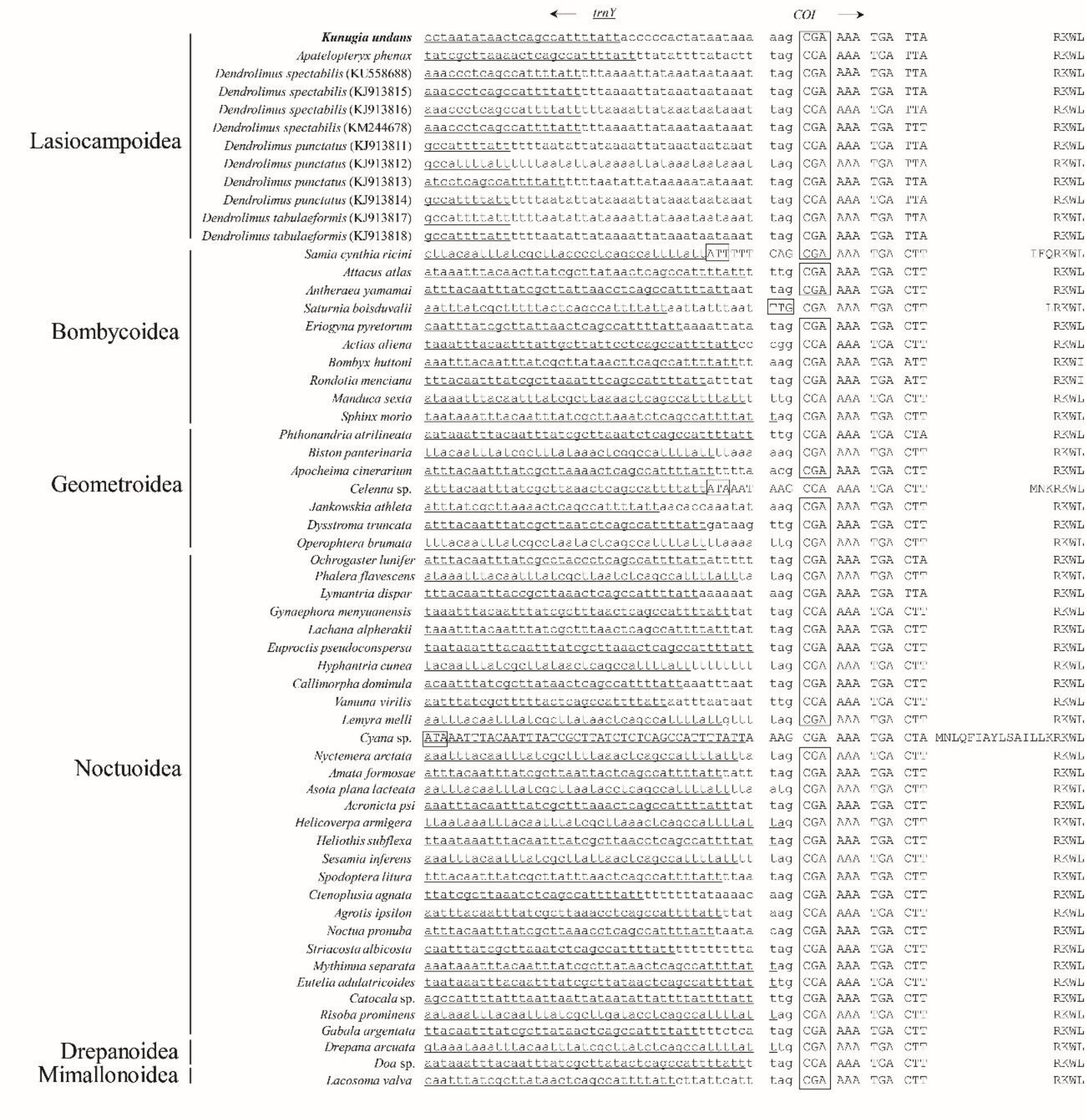

Figure S2 - Alignment of the initiation context of the Macroheterocera *COI*. The first 4–19 codons are shown on the right. Underlined nucleotides indicate the adjacent partial *trnY* sequence. Arrows indicate the transcriptional direction, and boxed nucleotides indicate currently proposed translation initiators.
