# Supplementary material for: Complete mitochondrial genome of the lappet moth, Kunugia undans (Lepidoptera: Lasiocampidae): genomic comparisons among macroheteroceran superfamilies
Source: Genet Mol Biol. 2017 Jul 31;40(3):717–23. doi: 10.1590/1678-4685-GMB-2016-0298 (PMC5596373; doi:10.1590/1678-4685-GMB-2016-0298)
Supplement: Supplementary file 6 [file 1415-4757-gmb-1678-4685-GMB-2016-0298-Suppl04.pdf]

Genetics and Molecular Biology, 40, 3 (2017)

Supplementary Material to "Complete mitochondrial genome of the lappet moth, Kunugia undans (Lepidoptera: Lasiocampidae): genomic comparisons among macroheteroceran superfamilies"

Table S4 - Frequency of the four most frequently used codons in Macroheterocera.

Species	Codon				Total
	TTA (L)	ATT (I)	TTT (F)	ATA (M)	
**Bombycoidea**					
Saturniidae					
Samia cynthia ricini	482/12.9	419/11.2	346/9.3	232/6.2	1479/39.6
Attacus atlas	443/11.9	407/10.9	353/9.5	252/6.8	1455/39.1
Antheraea yamamai	462/12.4	430/11.5	367/9.8	247/6.6	1506/40.3
Saturnia boisduvalii	463/12.4	430/11.5	376/10.1	250/6.7	1519/40.7
Eriogyna pyretorum	462/12.4	429/11.5	367/9.8	262/7.0	1520/40.7
Actias aliena	430/11.5	399/10.7	338/9.1	224/6.0	1391/37.3
Bombycidae					
Bombyx huttoni	458/12.3	416/11.2	387/10.4	276/7.4	1537/41.3
Rondotia menciana	443/11.9	395/10.6	340/9.1	248/6.6	1426/38.2
Sphingidae					
Manduca sexta	487/13.1	439/11.8	358/9.6	285/7.7	1569/42.2
Sphinx morio	496/13.3	431/11.6	350/9.4	278/7.5	1555/41.8
**Geometroidea**					
Geometridae					
Phthonandria atrilineata	495/13.3	415/11.1	338/9.1	257/6.9	1505/40.4
Biston panterinaria	437/11.7	393/10.5	302/8.1	267/7.2	1399/37.5
Apocheima cinerarium	469/12.6	421/11.3	334/8.9	266/7.1	1490/39.9
Celenna sp.	483/13.0	431/11.6	320/8.6	247/6.6	1481/39.8
Jankowskia athleta	450/12.1	414/11.1	325/8.7	240/6.4	1429/38.3
Dysstroma truncata	432/11.6	416/11.2	366/9.8	259/7.0	1473/39.6
Operophtera brumata	439/11.8	417/11.2	338/9.1	256/6.9	1450/39.0
**Noctuoidea**					
Notodontidae					
Ochrogaster lunifer	392/10.5	379/10.1	302/8.1	241/6.4	1314/35.1
Phalera flavescens	473/12.7	437/11.7	339/9.1	263/7.1	1512/40.6
Erebidae					
Lymantria dispar	448/12.0	408/10.9	327/8.8	249/6.7	1432/38.4
Gynaephora menyuanensis	446/12.0	455/12.2	340/9.1	298/8.0	1539/41.3
Lachana alpherakii	446/12.0	450/12.1	336/9.0	296/7.9	1528/41.0
Euproctis pseudoconspersa	425/11.4	421/11.3	318/8.6	259/7.0	1423/38.3
Hyphantria cunea	449/12.1	413/11.1	332/8.9	268/7.2	1462/39.3
Callimorpha dominula	472/12.7	468/12.6	334/9.0	273/7.3	1547/41.6
Vamuna virilis	471/12.6	421/11.3	338/9.1	242/6.5	1472/39.5
Lemyra melli	425/11.4	412/11.1	327/8.8	248/6.7	1412/38.0
Cyana sp.	479/12.8	433/11.6	335/9.0	267/7.2	1514/40.6
Nyctemera arctata	460/12.3	450/12.1	343/9.2	268/7.2	1521/40.8
Amata formosae	430/11.5	411/11.0	342/9.2	269/7.2	1452/38.9
Asota plana lacteata	455/12.2	424/11.4	320/8.6	266/7.1	1465/39.3
Noctuidae					
Acronicta psi	436/11.7	408/10.8	334/9.0	242/6.5	1420/38.0
Helicoverpa armigera	476/12.8	438/11.8	338/9.1	263/7.1	1515/40.8
Heliothis subflexa	462/12.4	446/12.0	335/9.0	270/7.3	1513/40.7
Sesamia inferens	463/12.4	425/11.4	355/9.5	240/6.4	1483/39.7
Spodoptera litura	484/13.0	431/11.6	334/9.0	269/7.2	1518/40.8

Species	Codon				Total
	TTA (L)	ATT (I)	TTT (F)	ATA (M)	
Ctenoplusia agnata	464/12.4	455/12.2	348/9.3	270/7.2	1537/41.1
Agrotis ipsilon	485/13.0	448/12.0	342/9.2	260/7.0	1535/41.2
Noctua pronuba	488/13.2	452/12.2	339/9.1	258/7.0	1537/44.6
Striacosta albicosta	453/12.2	414/11.1	327/8.8	232/6.2	1426/38.3
Mythimna separata	472/12.7	450/12.1	343/9.2	257/6.9	1522/40.9
Eutelia adulatricoides	473/12.7	411/11.0	348/9.3	252/6.8	1484/39.8
Catocala sp.	447/12.0	419/11.2	351/9.4	310/8.3	1527/40.9
Nolidae					
Risoba prominens	504/13.5	431/12.2	348/9.4	252/6.8	1535/41.9
Gabala argentata	503/12.0	456/12.2	349/9.1	252/8.0	1560/41.3
**Drepanoidea**					
Drepanidae					
Drepana arcuata	453/12.2	441/11.9	335/9.0	284/7.7	1513/40.8
Doidae					
Doa sp.	461/12.5	431/11.6	364/9.8	260/7.0	1516/40.9
**Mimallonoidea**					
Mimallonidae					
Lacosoma valva	450/12.1	414/11.1	329/8.8	272/7.3	1465/39.3
Average	460/12.3	426/11.4	341/9.1	260/7.0	1487/39.9

The corresponding amino acids are indicated in parentheses. Values after the backslash indicate the percentage of corresponding codons.
