# Supplementary material for: Complete mitochondrial genome of the lappet moth, Kunugia undans (Lepidoptera: Lasiocampidae): genomic comparisons among macroheteroceran superfamilies
Source: Genet Mol Biol. 2017 Jul 31;40(3):717–23. doi: 10.1590/1678-4685-GMB-2016-0298 (PMC5596373; doi:10.1590/1678-4685-GMB-2016-0298)
Supplement: Supplementary file 8 [file 1415-4757-gmb-1678-4685-GMB-2016-0298-Suppl08.pdf]

Supplementary Material to "Complete mitochondrial genome of the lappet moth, Kunugia undans (Lepidoptera: Lasiocampidae): genomic comparisons among macroheteroceran superfamilies"

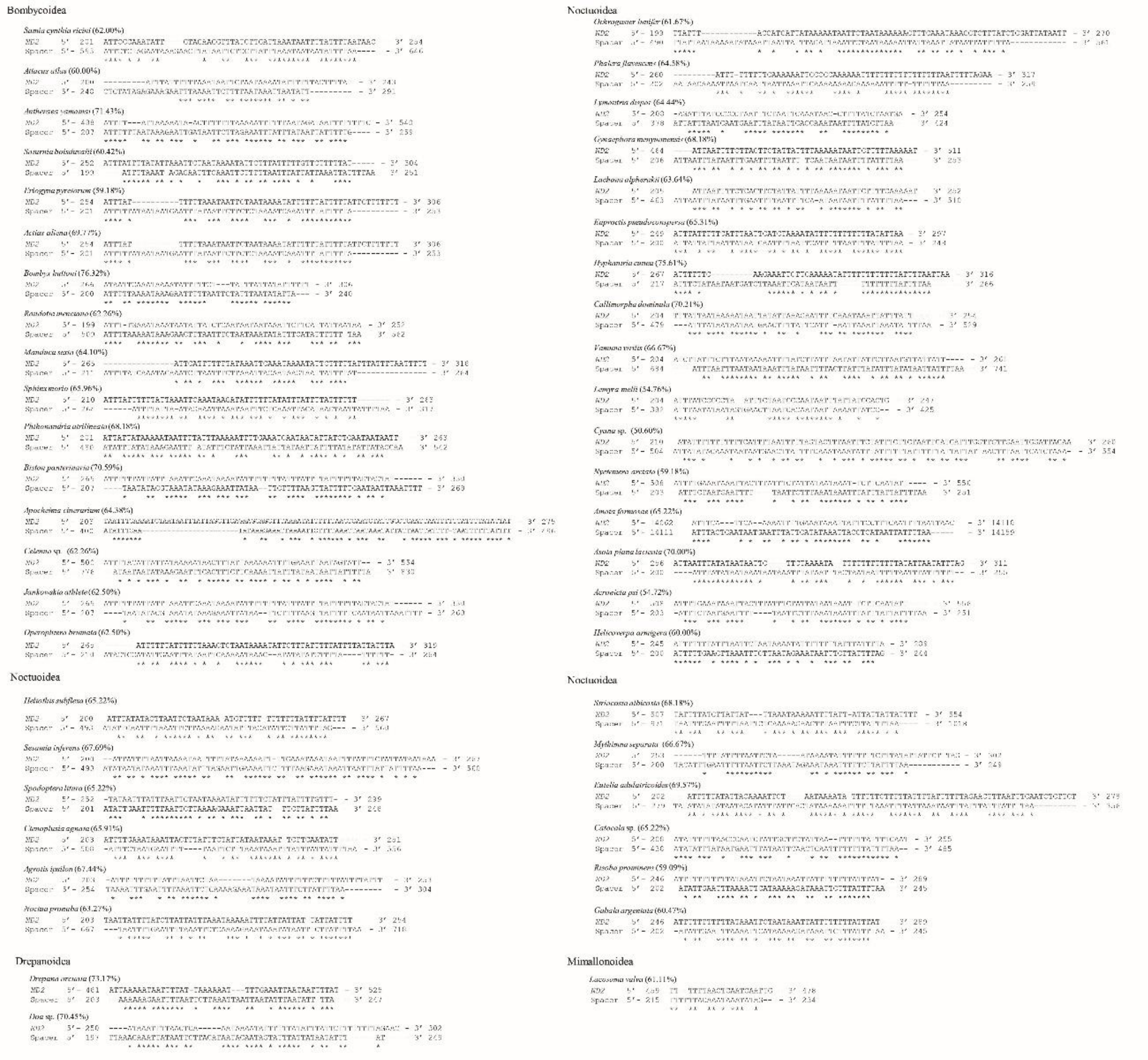

Figure S4 - Alignment of the spacer sequence (located between *trnQ* and *ND2*) and neighboring partial Macroheterocera *ND2*. Asterisks indicate consensus sequences in the alignment. Sequence homology between the spacer and the *ND2* is shown in the parentheses next to the species name. The beginning and end nucleotide positions of the sequences are indicated.
