# Supplementary material for: Complete mitochondrial genome of the lappet moth, Kunugia undans (Lepidoptera: Lasiocampidae): genomic comparisons among macroheteroceran superfamilies
Source: Genet Mol Biol. 2017 Jul 31;40(3):717–23. doi: 10.1590/1678-4685-GMB-2016-0298 (PMC5596373; doi:10.1590/1678-4685-GMB-2016-0298)
Supplement: Supplementary file 9 [file 1415-4757-gmb-1678-4685-GMB-2016-0298-Suppl09.pdf]

Genetics and Molecular Biology, 40, 3 (2017)

Supplementary Material to "Complete mitochondrial genome of the lappet moth, Kunugia undans (Lepidoptera: Lasiocampidae): genomic comparisons among macroheteroceran superfamilies"

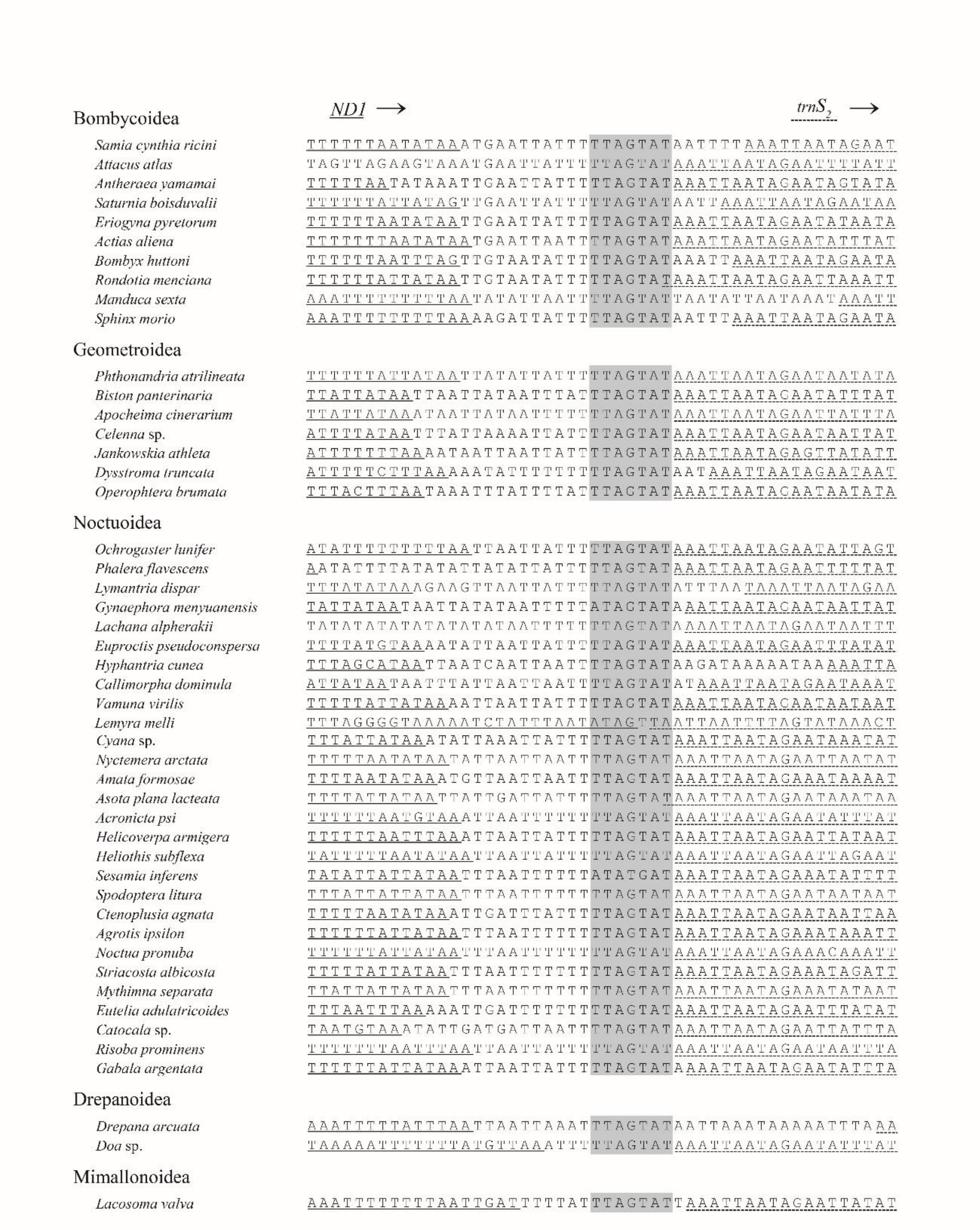

Figure S5 - Alignment of the internal spacer sequence located between *ND1* and *trnS2* of Macroheterocera. The shaded nucleotides indicate the conserved heptanucleotide (TTAGTAT) region. Underlined nucleotides indicate the adjacent partial sequences of *ND1* and *trnS2*. Arrows indicate the transcriptional direction.
