# Supplementary material for: Complete mitochondrial genome of the lappet moth, Kunugia undans (Lepidoptera: Lasiocampidae): genomic comparisons among macroheteroceran superfamilies
Source: Genet Mol Biol. 2017 Jul 31;40(3):717–23. doi: 10.1590/1678-4685-GMB-2016-0298 (PMC5596373; doi:10.1590/1678-4685-GMB-2016-0298)
Supplement: Supplementary file 7 [file 1415-4757-gmb-1678-4685-GMB-2016-0298-Suppl07.pdf]

Supplementary Material to "Complete mitochondrial genome of the lappet moth, Kunugia undans (Lepidoptera: Lasiocampidae): genomic comparisons among macroheteroceran superfamilies"

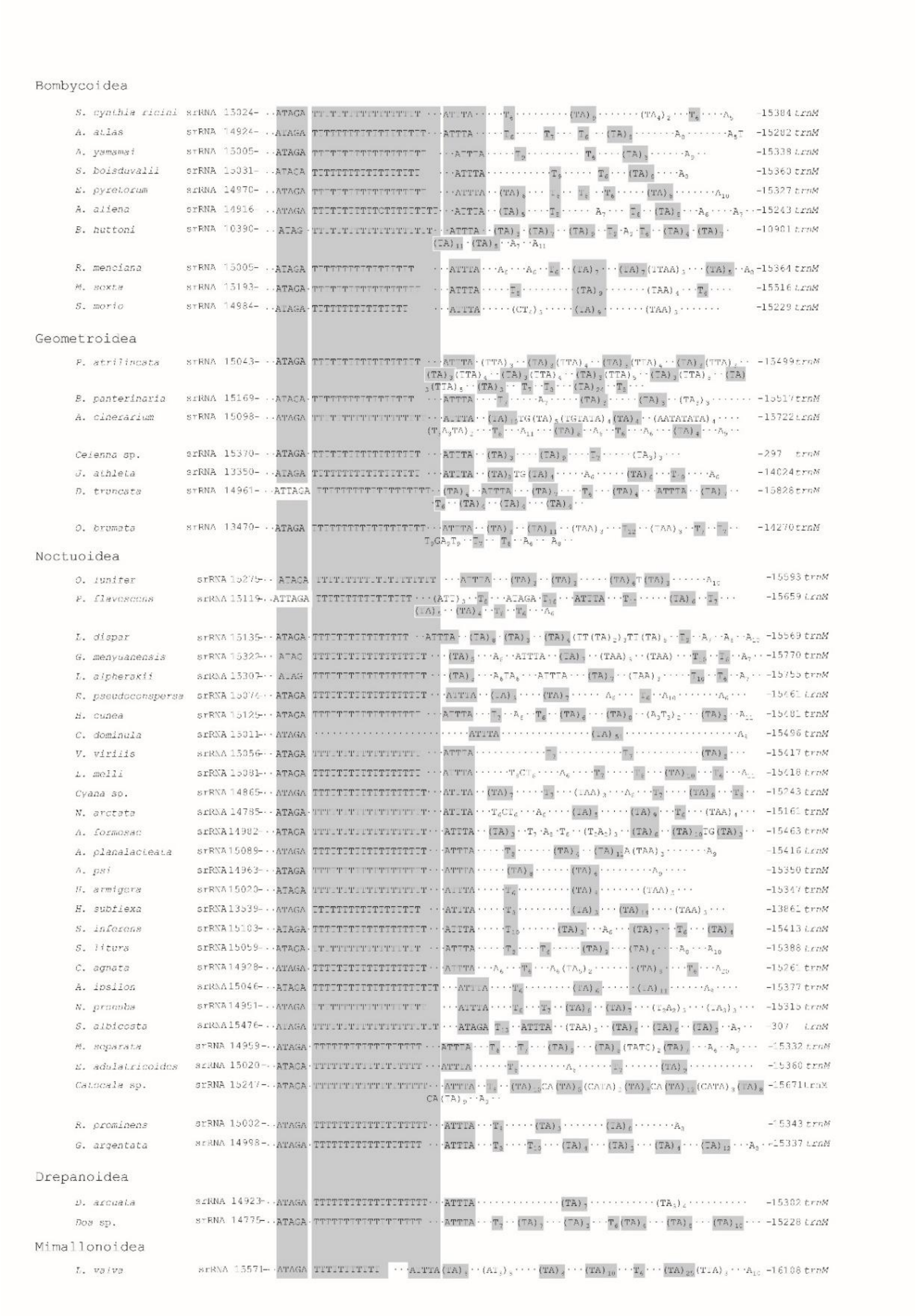

Figure S3 - Schematic illustration of the A+T-rich region of Macroheterocera. The shaded nucleotides indicate conserved sequences such as the ATAGA motif, poly-T stretch, ATTTA sequence, and microsatellite-like TA repeat sequences. Dots between sequences indicate omitted sequences, and arrows indicate the transcriptional direction. Subscripts indicate the repeat number. GenBank accession numbers for each species are presented in Table S2.
